# Signal production and detection specificity in *Vibrio* CqsA/CqsS quorum-sensing systems

**DOI:** 10.1111/j.1365-2958.2011.07548.x

**Published:** 2011-01-26

**Authors:** Wai-Leung Ng, Lark J Perez, Yunzhou Wei, Christina Kraml, Martin F Semmelhack, Bonnie L Bassler

**Affiliations:** 1 Departments of Molecular Biology; 2 Chemistry, Princeton UniversityPrinceton, NJ, USA; 3 Lotus Separations LLCPrinceton, NJ, USA; 4 Howard Hughes Medical InstituteChevy Chase, MD, USA

## Abstract

Quorum sensing is a process of bacterial cell–cell communication that enables populations of cells to carry out behaviours in unison. Quorum sensing involves detection of the density-dependent accumulation of extracellular signal molecules called autoinducers that elicit population-wide changes in gene expression. In *Vibrio* species, CqsS is a membrane-bound histidine kinase that acts as the receptor for the CAI-1 autoinducer which is produced by the CqsA synthase. In *Vibrio cholerae*, CAI-1 is (*S*)-3-hydroxytridecan-4-one. The C170 residue of *V. cholerae* CqsS specifies a preference for a ligand with a 10-carbon tail length. However, a phenylalanine is present at this position in *Vibrio harveyi* CqsS and other homologues, suggesting that a shorter CAI-1-like molecule functions as the signal. To investigate this, we purified the *V. harveyi* CqsS ligand, and determined that it is (*Z*)-3-aminoundec-2-en-4-one (Ea-C8-CAI-1) carrying an 8-carbon tail. The *V. harveyi* CqsA/CqsS system is exquisitely selective for production and detection of this ligand, while the *V. cholerae* CqsA/CqsS counterparts show relaxed specificity in both production and detection. We isolated CqsS mutants in each species that display reversed specificity for ligands. Our analysis provides insight into how fidelity is maintained in signal transduction systems.

## Introduction

Many bacteria produce and release extracellular signalling molecules called autoinducers. By monitoring the accumulation of autoinducers, bacteria track changes in population density and species complexity in the vicinity. This process, known as quorum sensing, enables groups of bacteria to synchronize gene expression and carry out collective behaviours such as bioluminescence, biofilm formation, competence and virulence factor production, which presumably are not productive when performed by a single bacterium (Fuqua and Greenberg, 2002; Pappas *et al*., 2004; Novick and Geisinger, 2008; Ng and Bassler, 2009; Williams and Camara, 2009).

**Fig. 1:**
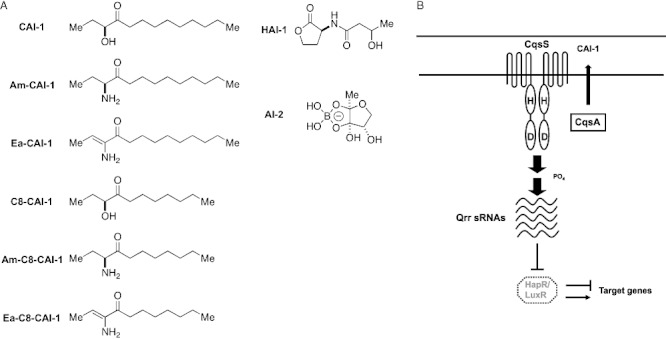
(A) Autoinducer molecules produced and detected by *Vibrio* species. Molecules in the left column are the CAI-1 type. HAI-1 and AI-2 are shown in the right column.B. The CqsA/CqsS quorum-sensing system of *Vibrio* species. Black arrows denote phosphate flow from the CqsS receptor to LuxO at low cell density. Under this condition, the Qrr sRNAs are transcribed and inhibit translation of *luxR* (*hapR*) transcripts. Thus, LuxR (HapR) proteins are not produced. At high cell density, in the presence of autoinducers, phosphate flow in the signal transduction pathway is reversed, the *qrr* genes are not transcribed, *luxR* (*hapR*) mRNA is translated, and LuxR (HapR) protein is produced, and it initiates the quorum-sensing response.

Many *Vibrio* species possess two or more quorum-sensing systems that channel multiple autoinducer inputs into the output response (Bassler *et al*., 1994; Miller *et al*., 2002; Henke and Bassler, 2004). For example, the human pathogen *Vibrio cholerae* activates quorum sensing in response to two different autoinducers, CAI-1 and AI-2 (Chen *et al*., 2002; Miller *et al*., 2002; Higgins *et al*., 2007) (Fig. 1A); whereas the marine bacterium *Vibrio harveyi* detects three distinct autoinducers, HAI-1, AI-2 and Vh-CAI-1 (Cao and Meighen, 1989; Chen *et al*., 2002; Henke and Bassler, 2004) (Fig. 1A). Presumably, bacteria extract unique information from each autoinducer. Consistent with this notion, HAI-1 (Fig. 1A), identified as 3-hydroxybutanoyl homoserine lactone, is produced by the LuxM synthase and detected by the LuxN receptor. The LuxM/LuxN system is present in *V. harveyi* and a few other very closely related *Vibrio* species, and HAI-1 is suggested to be used for intra-species communication (Cao and Meighen, 1989; Bassler *et al*., 1993; 1994; Henke and Bassler, 2004). AI-2, identified as (2*S*, 4*S*)-2-methyl-2,3,3,4-tetrahydroxytetrahydrofuran borate in *Vibrios*, is produced by the LuxS enzyme and detected by the LuxPQ receptor (Bassler *et al*., 1994; 1997; Schauder *et al*., 2001; Chen *et al*., 2002; Neiditch *et al*., 2005). LuxS is present in many bacterial species; thus, AI-2 is considered a signal for inter-species communication (Xavier and Bassler, 2003; Vendeville *et al*., 2005; Federle, 2009). CAI-1, identified in *V. cholerae* as (*S*)-3-hydroxytridecan-4-one, is produced by the CqsA synthase and detected by the CqsS receptor. The CqsA/CqsS system is conserved in many *Vibrio* species (Miller *et al*., 2002; Henke and Bassler, 2004; Higgins *et al*., 2007), suggesting it may be used for communication between *Vibrios*. The structure of the CAI-1 signal produced by *V. harveyi* (Vh-CAI-1) has not been examined.

The information contained in the three autoinducers in *V. harveyi* (two in *V. cholerae*) is shuttled into a common phosphorelay signal transduction pathway (Fig. 1B shows the CqsA/CqsS system as an example). Collectively these autoinducers activate production of the master quorum-sensing regulator LuxR (HapR in *V. cholerae*). LuxR (HapR) acts as both a transcriptional activator and a transcriptional repressor. In *V. harveyi*, LuxR activates genes for bioluminescence and regulates at least 50 additional targets (Showalter *et al*., 1990; Waters and Bassler, 2006; Pompeani *et al*., 2008). In *V. cholerae*, HapR activates competence genes and HapA protease production, and represses virulence and biofilm formation (Kovacikova and Skorupski, 2002; Zhu *et al*., 2002; Hammer and Bassler, 2003; 2009; Zhu and Mekalanos, 2003; Blokesch and Schoolnik, 2008; Tsou *et al*., 2009).

Specificity in ligand–receptor interactions presumably plays a role in preventing cross-talk between related signals and eliminating noise from molecules similar to autoinducers that exist in the environment. However, some quorum-sensing systems display low signal discrimination and multiple autoinducers can activate these circuits (McClean *et al*., 1997; Cha *et al*., 1998). The CqsA/CqsS system belongs to the latter class. Specifically, spent culture fluids from a variety of *Vibrio* species trigger a quorum-sensing response in a *V. cholerae* CAI-1 reporter strain (Henke and Bassler, 2004). This finding has been interpreted to mean that CAI-1 is used for inter-*Vibrio* cell–cell communication.

Strikingly, however, examination of *V. cholerae* CqsS receptor mutants displaying altered responses to natural and synthetic CAI-1 analogues showed that the CqsS residue Cys 170 imparts a preference for a CAI-1 molecule that carries a 10-carbon hydrocarbon tail (Ng *et al*., 2010). Substituting residue 170 with bulky aromatic amino acids such as phenylalanine or tyrosine (C170F or C170Y) results in a receptor mutant that only recognizes a CAI-1 analogue carrying an 8-carbon tail (C8-CAI-1, Fig. 1A). Examination of conservation among different CqsS homologues shows that residue 170 is substituted with a phenylalanine in *V. harveyi* CqsS and other homologues. A polymorphism at this particular position in CqsS suggests that a CAI-1 molecule with a 10-carbon tail is not likely to be the autoinducer detected by all CqsS receptors.

In this study, through purification and chemical synthesis, we identify the *V. harveyi* CqsS ligand as (*Z*)-3-aminoundec-2-en-4-one (Ea-C8-CAI-1, Fig. 1A). Ea-C8-CAI-1 is also produced and detected by *V. cholerae* CqsA/CqsS. Although the CqsA/CqsS systems in *V. harveyi* and *V. cholerae* are highly conserved, the *V. harveyi* CqsA synthase is highly selective for its substrate octanoyl CoA, and the *V. harveyi* CqsS receptor likewise displays an exquisite specificity for its ligand Ea-C8-CAI-1. In contrast, *V. cholerae* CqsA has less substrate specificity, accepting both decanoyl CoA and octanoyl CoA. The *V. cholerae* CqsS receptor is similarly less stringent and does not discriminate well between ligands. We isolated *V. cholerae* and *V. harveyi* CqsS mutants displaying increased and decreased ligand specificity respectively. We propose that the CqsA/CqsS systems in these two organisms have diverged from a common ancestral origin, and that differences arose from selective forces that favoured decreased specificity in *V. cholerae* and/or increased specificity in *V. harveyi.*

## Results

### *V. harveyi* CqsS detects a different ligand than does *V. cholerae* CqsS

**Fig. 2:**
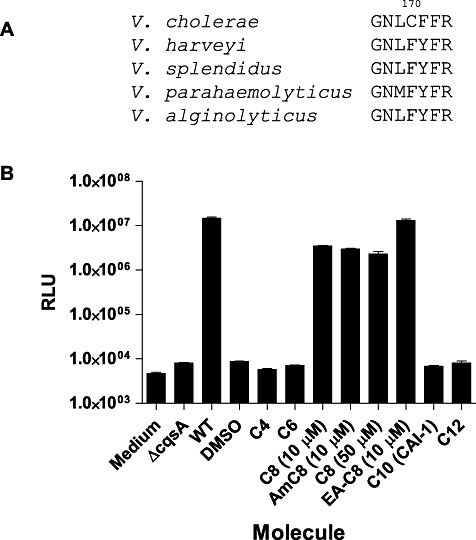
(A) Polymorphism in the CqsS receptors of *Vibrio* species. The C170 residue in *V. cholerae* is substituted with phenylalanine in multiple *Vibrio* species including *V. harveyi*.B. The *V. harveyi* CqsS-dependent quorum-sensing response to various CAI-1 type molecules. Activation of bioluminescence expression was measured in *V. harveyi* strain JMH626 in the presence of exogenously added CAI-1 type molecules. Δ*cqsA* and WT denote cell-free culture fluids prepared from a *V. harveyi* mutant lacking or containing the CqsA synthase respectively. See Fig. 1A for the structures of the different molecules. RLU denotes relative light units which is Lux/OD_600_.

The CqsS receptor is conserved among many *Vibrio* species. Notably, the transmembrane ligand-sensing domains share more than 70% amino acid sequence identity. This high sequence identity suggests that CqsS receptors could recognize a common or a related set of signalling molecules. (*S*)-3-hydroxytridecan-4-one and (*S*)-3-aminotridecan-4-one (i.e. CAI-1 and Am-CAI-1, respectively, Fig. 1A) are two potent 13-carbon CqsS agonists produced and detected by *V. cholerae* (Higgins *et al*., 2007; Kelly *et al*., 2009). We previously showed that a single amino acid change in the CqsS-sensing domain results in an altered ligand specificity (Ng *et al*., 2010). Specifically, replacing Cys 170 with a bulky amino acid causes a loss in response to CAI-1 and an increase in sensitivity to a CAI-1 analogue carrying a shortened 8-carbon tail (C8-CAI-1, Fig. 1A) (Ng *et al*., 2010). We inspected all CqsS receptor homologues in sequenced *Vibrios* and found that many of them, including the *V. harveyi* CqsS, carry a phenylalanine substitution at this particular position (Fig. 2A). Therefore, we suspect that these receptors must detect molecules that are shorter than CAI-1.

We chose *V. harveyi* as the model to examine the molecules detected by these naturally occurring polymorphic CqsS receptors. To do this, we measured light production in a *V. harveyi* reporter strain that responds exclusively to exogenously supplied CqsS ligands (JMH626, see *Experimental procedures*). Sterile medium and spent culture fluid from a Δ*cqsA* strain do not induce light production in JMH626. However, addition of 10% (v/v) spent culture fluid prepared from wild-type *V. harveyi* induces light production 3000-fold in JMH626 (Fig. 2B). Presumably, this is due to production of the endogenous CqsS ligand. We tested purified CAI-1 and CAI-1 analogues carrying different hydrocarbon tail lengths for stimulation of light production in this assay. CAI-1 analogues with 4-, 6-, 10- and 12-carbon tails did not stimulate light production (Fig. 2B). However, C8-CAI-1 (10 µM) and Am-C8-CAI-1 (10 µM) induce an approximately 400-fold increases in light production (Fig. 2B). Increasing the concentration to 50 µM did not further activate light production (Fig. 2B). Combining C8-CAI-1 with spent culture medium from a *V. harveyi*Δ*cqsA* strain also did not enhance induction (data not shown). These results suggest that *V. harveyi* CqsS does not detect the *V. cholerae* CAI-1 molecule (10-carbon tail) as an agonist, and that C8-CAI-1 is only a weak agonist for *V. harveyi* CqsS since it cannot induce maximal light production even at very high concentrations. We therefore reason that *V. harveyi* must produce a CAI-1 like molecule that likely has an 8-carbon tail with additional modification(s) that do not exist in CAI-1 or in C8-CAI-1.

In order to identify the molecule detected by the *V. harveyi* CqsS receptor, we overexpressed the *V. harveyi cqsA* gene in *Escherichia coli* (strain WN1327). Spent culture fluid (10% v/v) from WN1327, but not from *E. coli* carrying the empty vector, induced light production in JMH626 to the maximal level (data not shown). Moreover, activity could be quantitatively extracted with dichloromethane (DCM), which allowed us to separate the activity from other components. Concentration of the DCM extract to complete dryness led to a significant loss in activity; therefore in all our analyses, we avoided steps that resulted in activity decreases by monitoring the activity in each step.

A variety of reverse and normal phase HPLC methods (*Supporting information*) were used to isolate fractions capable of inducing maximal light production in *V. harveyi* JMH626. The purity of the active fractions was determined by ESI-TOF HRMS. One fraction that could induce maximal light production in JMH626 possessed a single ion peak with m/z [M+H^+^] of 184.1699, corresponding to a molecular formula of C_11_H_21_NO. A second fraction was weakly active and possessed a molecular ion peak with m/z [M+H^+^] of 187.1694, corresponding to a molecular formula of C_11_H_22_O_2_. Concentration of this second fraction allowed us to use NMR to determine that it contains 3-hydroxyundecan-4-one (C8-CAI-1) and its regioisomer 4-hydroxyundecan-3-one in approximately a 1:1 ratio. Through chemical synthesis and consistent with the above results, we found that C8-CAI-1 is only a weak agonist for the *V. harveyi* CqsS receptor. The regioisomer, 4-hydroxyundecan-3-one, is inactive (Fig. 2B and data not shown).

Direct characterization of the structure of the 184.1699 molecular ion by NMR was impossible because, as mentioned, concentration caused a complete loss in activity. We hypothesized that this molecule must have a structure similar to known CqsS agonists including CAI-1, Am-CAI-1 and C8-CAI-1. Based on its molecular formula C_11_H_21_NO, we predicted that the active molecule was likely a derivative of Am-C8-CAI-1 (Fig. 1A) possessing an additional degree of unsaturation. To identify the position of the unsaturation, we used super-critical fluid chromatography (SFC) to obtain a highly concentrated fraction of our desired molecule dissolved in a solvent suitable for direct NMR analysis (see *Experimental procedures*). The resulting maximally active fraction contains a molecule with a single olefinic proton by ^1^H-NMR (δ 5.77, q, *J* = 7.1 Hz) (*Supporting information*). Taken together with the molecular formula, the splitting pattern and chemical shift of this signal allows us to propose that the structure of the highly active *V. harveyi* CqsS agonist is (*Z*)-3-aminoundec-2-en-4-one (Ea-C8-CAI-1, Fig. 1A). We prepared Ea-C8-CAI-1 by total synthesis (*Supporting information*) and determined that, by ^1^H-NMR, the synthetic material contains the diagnostic olefinic quartet observed in the natural sample. Additionally, the synthetic compound has identical activity to the ligand-containing spent culture fluid harvested from wild-type *V. harveyi*; that is, they both induce maximal light production in JMH626 (Fig. 2B). We conclude the *V. harveyi* CqsS receptor detects Ea-C8-CAI-1 as its native ligand.

### Different *Vibrio* species display unique production and detection profiles for the CAI-1 family of molecules

Spent culture fluids from *V. harveyi* and *V. cholerae* cross-stimulate the CqsS-dependent quorum-sensing response of one another (Henke and Bassler, 2004) (data not shown). Because *V. harveyi* CqsS is insensitive to CAI-1 (10-carbon tail) and quite sensitive to Ea-C8-CAI-1, we hypothesized that *V. cholerae* must also produce and detect Ea-C8-CAI-1. Based on the above results, we also wondered if *V. cholerae* produces an enamino molecule (perhaps Ea-CAI-1, 10-carbon tail, Fig. 1A) analogous to Ea-C8-CAI-1 that promotes the response we observed in *V. harveyi*.

To examine these possibilities, we determined how much of each CAI-1 type molecule is produced by wild-type *V. harveyi* and *V. cholerae*. Both species were grown to early stationary phase and cell-free spent culture fluids were prepared (see *Experimental procedures*). CAI-1 type molecules were extracted with DCM for HRMS analysis. The concentration of each CAI-1 type molecule was calculated by comparison to the intensity of each ion peak to that of d_2_-CAI-1 (double deuterium labelled CAI-1), which was added as an internal standard at 500 nM prior to the extraction. Correction for the different ionization potentials of each of the CAI-1 type molecules was achieved using a mixed sample containing a known amount of each of the molecules (see *Experimental procedures*).

**Fig. 3:**
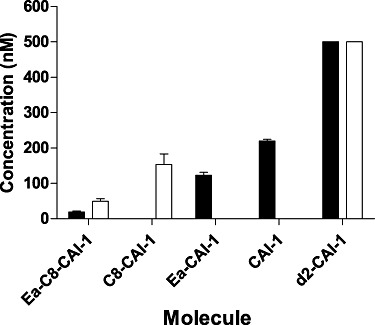
CAI-1 type molecule biosynthetic profiles for *V. cholerae* and *V. harveyi*. The concentrations of different CAI-1 type molecules present in cell-free culture fluids prepared from *V. cholerae* (black bars) and *V. harveyi* (white bars) were determined by HRMS analyses using d_2_-CAI-1 as the internal reference (final set of bars). Results from three independent replicates were averaged. Error bars denote standard deviations. See Fig. 1A for the structures of the different molecules.

In the *V. cholerae* spent culture fluids, we detected the molecular ions from Ea-C8-CAI-1, Ea-CAI-1 and CAI-1 at an average concentration of 19 nM, 139 nM and 222 nM respectively. We did not detect any C8-CAI-1 ion in the *V. cholerae* sample (Fig. 3). In the *V. harveyi* spent culture fluids, we only detected molecular ions from Ea-C8-CAI-1 and C8-CAI-1 at an average concentration of 50 nM and 154 nM respectively. No ion from CAI-1 or Ea-CAI-1 was detected (Fig. 3). The detection limits for Ea-C8-CAI-1, C8-CAI-1, Ea-CAI-1 and CAI-1 are 6.25 nM, 25 nM, 12.5 nM and 25 nM respectively (*Supporting information*).

**Fig. 4:**
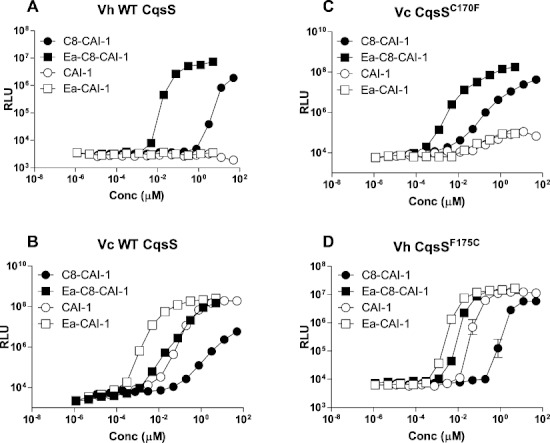
Response of *V. cholerae* and *V. harveyi* wild-type CqsS and CqsS mutants to different CAI-1 type molecules. The CqsS-dependent quorum-sensing responses to different CAI-1 type molecules were measured by assaying activation of bioluminescence expression in wild-type *V. harveyi* (A), wild-type *V. cholerae* (B), *V. cholerae* carrying CqsS C170F (C) and *V. harveyi* carrying CqsS F175C (D). C8-CAI-1, closed circles; Ea-C8-CAI-1, closed squares; CAI-1, open circles, Ea-CAI-1, open squares. See Fig. 1A for the structures of the different molecules. Representative data from at least three independent experiments are shown. See Table 1 for EC_50_ values for each receptor/ligand combination. RLU denotes relative light units.

We systematically analysed the relative activities of all of the CAI-1-related molecules observed in our HRMS analysis (i.e. C8-CAI-1, Ea-C8-CAI-1, CAI-1 and Ea-CAI-1) on *V. harveyi* and *V. cholerae* (Fig. 4, Table 1). As in the above experiments, we used JMH626 for the *V. harveyi* test. For *V. cholerae*, we used a mutant (WN1102, see *Experimental procedures*) that carries the *V. harveyi luxCDABE* operon (luciferase) and responds exclusively to exogenously supplied CqsS ligands. The *V. harveyi* wild-type CqsS receptor is highly sensitive to synthetic Ea-C8-CAI-1 (EC_50_ = 24 nM, EC_50_ is defined as the concentration of autoinducer that induces light production to half the maximum level) but only responds weakly to C8-CAI-1 (EC_50_ = 5 µM). The *V. harveyi* CqsS receptor is insensitive to Ea-CAI-1 and CAI-1 (Fig. 4A, Table 1). In contrast, the *V. cholerae* CqsS receptor responds to a wider array of molecules including Ea-C8-CAI-1 (EC_50_ = 36 nM), Ea-CAI-1 (EC_50_ = 4 nM) and CAI-1 (EC_50_ = 62 nM), but is only weakly sensitive to C8-CAI-1 (EC_50_ = 1.2 µM) (Fig. 4B and Table 1). We also tested Am-CAI-1 and Am-C8-CAI-1; their potencies are comparable to the CAI-1 and C8-CAI-1 counterparts in each CqsS receptor (data not shown).

**Table 1:** CqsS receptor sensitivities to CAI-1 type molecules.^a^

	EC_50_ (nM)			
CqsS	C8-CAI-1	Ea-C8-CAI-1	CAI-1	Ea-CAI-1
Vc WT	1175 ± 300	36 ± 8	62 ± 12	4 ± 3
Vc C170F	268 ± 91	3 ± 0.4	NA	NA
Vh WT	5294 ± 967	24 ± 7	NA	NA
Vh F175C	3177 ± 219	12 ± 3	36 ± 8	6 ± 4

a Numbers are shown as mean ± standard error of mean.

NA, not applicable.

Based on these results, we propose that, in the growth conditions used on this study, wild-type *V. harveyi* only detects Ea-C8-CAI-1 through CqsS to activate quorum sensing. Although C8-CAI-1 is a weak agonist, the EC_50_ value is significantly larger than the concentration of C8-CAI-1 released by *V. harveyi*. Using the same reasoning, *V. cholerae* likely detects three molecules, Ea-CAI-1, CAI-1 and Ea-C8-CAI-1, but not C8-CAI-1. Therefore, *V. harveyi* CqsS has a stringent specificity for its ligand, while *V. cholerae* CqsS is somewhat promiscuous. In addition, our results show that there is a common molecule, Ea-C8-CAI-1, produced and detected efficiently by both *V. cholerae* and *V. harveyi* CqsS (Fig. 4 and Table 1). We propose that Ea-C8-CAI-1 is the chemical signal that is used for inter-*Vibrio* cell–cell communication.

### CqsA substrate specificity correlates with the CAI-1 biosynthetic profile

In another study, we showed that *V. cholerae* CAI-1 is derived from the precursor Ea-CAI-1, which is synthesized by *V. cholerae* CqsA using *S*-adenosylmethionine (SAM) and decanoyl coenzyme A (C10-CoA) as substrates (Y. Wei *et al*., 2011). Likewise, in *V. harveyi*, C8-CAI-1 is derived from Ea-C8-CAI-1, which is synthesized by CqsA from SAM and octanoyl CoA (C8-CoA) (*Supporting information*). However, these findings do not account for all the CAI-1 type molecules we detect in cell-free culture fluids. Specifically, *V. cholerae* must also produce Ea-C8-CAI-1. We wondered therefore if similar to their cognate CqsS receptors, *V. harveyi* and *V. cholerae* CqsA enzymes possess high and low substrate specificity respectively. To examine this possibility, the two CqsA enzymes were purified and used in a coupled-enzyme assay (*Experimental procedures*) to determine the kinetic parameters with respect to processing C8-CoA or C10-CoA as substrates.

*Vibrio cholerae* CqsA uses C10-CoA and C8-CoA to make Ea-CAI-1 and Ea-C8-CAI-1 respectively (Table 2). However, as indicated by the differences in both *K*_m_ and *k*_cat_, *V. cholerae* CqsA prefers C10-CoA over C8-CoA as substrate (four- to sixfold). In contrast, the *V. harveyi* CqsA enzyme only uses C8-CoA to produce Ea-C8-CAI-1 and is unable to use C10-CoA as a substrate (Table 2). Thus, the unique preferences of the CqsA enzymes for their acyl CoA substrates match the *in vivo* CAI-1 production profiles displayed by *V. cholerae* and *V. harveyi*. Interestingly, the CqsA substrate preferences also highly correlate with the CqsS ligand selectivities. We propose that the CqsA enzymes and the CqsS receptors coevolved to ensure productive ligand–receptor interactions and high-fidelity signalling.

**Table 2:** Kinetic parameters for *V. cholerae* and *V. harveyi* CqsA enzymes.

Enzyme Substrate	Vh CqsA C8 CoA	Vh CqsA C10 CoA	Vc CqsA C10 CoA	Vc CqsA C8 CoA
*K*_m_ (µM)	14.1 ± 1.3	ND	13.0 ± 3.1	73 ± 4.9
*V*_max_ (µM min^−1^)	0.64 ± 0.02	ND	1.2 ± 0.15	1.4 ± 0.05
*k*_cat_ (min^−1^)	10.7 ± 0.4	ND	19.5 ± 2.5	4.5 ± 0.17

ND, not detected.

### CqsS amino acid residues responsible for ligand specificity

The *V. harveyi* receptor shows a strong preference for CAI-1 molecules carrying 8-carbon tails (Fig. 4A, Table 1). In addition, the presence of the enamino group in C8-Ea-CAI-1 is crucial for its agonist activity for the *V. harveyi* receptor (Fig. 4A, Table 1). In contrast, *V. cholerae* CqsS is promiscuous and detects both 8-carbon and 10-carbon tailed molecules. The presence of the enamino group is not obligatory for agonist activity in *V. cholerae* (Fig. 4B, Table 1). To understand ligand preference, we used mutagenesis to pinpoint residues that are essential for detection of the different features of the ligands (i.e. chain length and presence of the enamino group).

First, we focus on chain length discrimination. We have previously performed an analysis on *V. cholerae* CqsS with two molecules, CAI-1 and C8-CAI-1(Ng *et al*., 2010). We now expand this analysis using all four molecules described here (i.e. CAI-1, C8-CAI-1, Ea-CAI-1 and Ea-C8-CAI-1), and both *V. cholerae* and *V. harveyi* CqsS receptors are studied. As mentioned, *V. cholerae* CqsS prefers CAI-1 over C8-CAI-1 due to the presence of Cys 170 (Ng *et al*., 2010). *V. cholerae* CqsS prefers Ea-CAI-1 over Ea-C8-CAI-1 (Fig. 4B). Consistent with this finding, *V. cholerae* CqsS^C170F^ only detects Ea-C8-CAI-1 and C8-CAI-1, but not CAI-1 and Ea-CAI-1 (Fig. 4C, Table 1). Likewise, we mutated the phenylalanine (F175) in the corresponding position in the *V. harveyi* CqsS receptor to cysteine. We found that the CqsS^F175C^ mutant exhibits relaxed specificity and detects both Ea-CAI-1 and CAI-1 as agonists (Fig. 4D, Table 1). In addition, and similar to wild-type *V. cholerae* CqsS, the *V. harveyi* CqsS^F175C^ mutant also detects Ea-C8-CAI-1 and is only weakly sensitive to C8-CAI-1 (Fig. 4D, Table 1). Moreover, the *V. harveyi* CqsS^F175C^ mutant is most sensitive to Ea-CAI-1. Thus, in terms of the chain length preference, the presence of a cysteine residue at position 170 decreases the overall specificity of the CqsS receptor, allowing detection of ligands with both 8- and 10-carbon tails. The presence of phenylalanine at this position increases the CqsS specificity for ligands that carry an 8-carbon tail.

Unexpectedly, detection of the enamino group also depends on the chain length of the molecule. Specifically, the enamino group is critical for activity only when it is present in a molecule carrying an 8-carbon tail (Fig. 4, Table 1). For example, there is a 10-fold difference in EC_50_ between CAI-1 and Ea-CAI-1 in *V. cholerae* CqsS and in the *V. harveyi* CqsS^F175C^ mutant. In contrast, there are 30-fold and 200-fold differences in EC_50_ between C8-CAI-1 and Ea-C8-CAI-1 in these two receptors (Table 1). Moreover, unlike CAI-1, C8-CAI-1 does not elicit a maximum quorum-sensing response in any CqsS receptor tested (Fig. 4, Table 1). These results suggest that C8-CAI-1 lacking the enamino group cannot properly interact with either the *V. cholerae* or the *V. harveyi* CqsS receptor. We have not been able to isolate any mutant that eliminates the requirement for the enamino group in Ea-C8-CAI-1. Thus, the molecular determinant for this preference remains unclear. One possibly is that the amino group forms a hydrogen bond with a backbone carbonyl in the receptor.

## Discussion

The CqsA/CqsS quorum-sensing system responds to molecules made by a variety of *Vibrio* species (Henke and Bassler, 2004), yet the identities of the active signalling molecules have not been well characterized. The first CAI-1 signal examined, that of *V. cholerae*, is (S)-3-hydroxytridecan-4-one (Higgins *et al*., 2007) (CAI-1, Fig. 1A). Here, we show that CAI-1 cannot be detected by the *V. harveyi* CqsS receptor due to an incompatibility between the 10-carbon tail in CAI-1 and the presence of a bulky F175 residue in the *V. harveyi* CqsS receptor. Using purification and total *in vitro* synthesis, we identified the ligand for the *V. harveyi* CqsS to be (*Z*)-3-aminoundec-2-en-4-one (Ea-C8-CAI-1, Fig. 1A). Ea-C8-CAI-1 fulfils the requirements for being an inter-*Vibrio* quorum-sensing signal because it is produced and detected by multiple *Vibrio* species (Figs 3 and 4, Table 1).

Although we focused in this study on *V. harveyi* and *V. cholerae* as test cases, the CqsS and CqsA sequences from most other *Vibrio* species can be readily categorized into one of the two classes represented by these examples. The *V. cholerae* system has relaxed specificity in both substrate selection by CqsA and ligand detection by CqsS (Figs 3 and 4, Tables 1 and 2). *V. cholerae* produces and detects three different molecules: CAI-1, Ea-CAI-1 and Ea-C8-CAI-1. In addition, the absence of an enamino group in CAI-1 does not significantly hamper its agonist activity. In contrast, the *V. harveyi* system is stringent in both CqsA-dependent production and CqsS-directed detection of the ligand (Figs 3 and 4, Tables 1 and 2). *V. harveyi* produces C8-CAI-1 and Ea-C8-CAI-1, but only Ea-C8-CAI-1 is detected. The presence of the enamino group is critical for ligand activity in *V. harveyi* (Figs 3 and 4, Tables 1 and 2). Based on these results, we suggest that CqsA/CqsS systems similar to the *V. harveyi* system are stringent, in which the unique substrate for CqsA is C8-CoA and the unique signal for CqsS is Ea-C8-CAI-1. Consistent with this prediction, we found that only Ea-C8-CAI-1 and C8-CAI-1, but not CAI-1 and Ea-CAI-1, are present in spent culture fluids prepared from several of these species (e.g. *Vibrio parahaemolyticus, Vibrio alginolyticus, Vibrio anguillarum* and *Vibrio furnissii*, data not shown). In contrast, CqsA/CqsS systems similar to the *V. cholerae* system are relaxed (e.g. *Vibrio mimicus* and some sequenced natural *Vibrio* isolates). Both C8-CoA and C10-CoA can be used as substrates for CqsA, and multiple signals including CAI-1, Ea-CAI-1 and Ea-C8-CAI-1 can be detected by CqsS. Ea-C8-CAI-1 links the two types of systems as it is produced and detected by both.

Ea-CAI-1 and Ea-C8-CAI-1 are both potent agonists for *V. cholerae* CqsS, and thus we wondered why we did not identify these enamino compounds previously (Higgins *et al*., 2007). We suspect that the enamino activities were destroyed during our previous sample preparation because our present results demonstrate that enamino molecules are labile following concentration or heat treatment. The procedures we used to initially isolate *V. cholerae* CAI-1 likely did not preserve the enamino molecules. However, because the *V. cholerae* CqsS receptor shows low stringency for the enamino group (Fig. 4, Table 1), we were able to identify CAI-1 as one of the *V. cholerae* CqsS ligands (Higgins *et al*., 2007). A detailed analysis of the mechanism by which Ea-CAI-1 is produced by CqsA and how Ea-CAI-1 is subsequently converted into CAI-1 is described elsewhere (Y. Wei *et al*., 2011).

In terms of signal specificity, the residue conferring the difference in receptor specificity is located in the final transmembrane helix of CqsS (Ng *et al*., 2010). Receptor specificity can be manipulated by incorporation of a single substitution at the C170 or F175 position of the CqsS receptor of *V. cholerae* or *V. harveyi* respectively. This polymorphism is particularly important for chain length preference. The presence of a small amino acid residue such as cysteine decreases the receptor selectivity, while the presence of a bulky amino acid residue such as phenylalanine increases selectivity (Fig. 4, Table 1).

The analogous mechanism underlying CqsA substrate preference is not understood. Based on the *V. cholerae* CqsA crystal structure, it is believed that the 10-carbon hydrocarbon chain sits in an enclosed hydrophobic pocket lined by H30, V32, F79, F257, I263, F264, C346, P348 and A349 (Jahan *et al*., 2009; Kelly *et al*., 2009). All of these residues are conserved in *V. harveyi* CqsA so they cannot be responsible for specifying the C8 substrate tail length preference. Additional biochemical and genetic studies are required to understand CqsA substrate specificity in *V. harveyi* or lack thereof in *V. cholerae*.

Although a few homologues of the CqsA/CqsS system are found in non-*Vibrio* species (see Tiaden *et al*., 2010a), to date, only one such system (i.e. the *Legionella pneumophila* LqsA/LqsS system) has been studied (Tiaden *et al*., 2007; 2008; 2010b; Spirig *et al*., 2008). An α-hydroxyketone molecule LAI-1 (3-hydroxypentadecan-4-one, that is, CAI-1 with a 12-carbon tail) is proposed to be the LqsS ligand. It is not known if the LqsA synthase uses a mechanism identical to CqsA to produce an enamino compound which is subsequently converted to LAI-1. The sequence homology is weak for the ligand–receptor interaction region in the sixth transmembrane helix of LqsS and CqsS; therefore, it is unclear how LqsS detects a ligand with a 12-carbon tail.

In Gram-negative bacteria, LuxI/LuxR quorum-sensing systems are commonly used. LuxI type proteins produce acyl homoserine lactones, which are detected by cognate cytoplasmic LuxR type receptors (Fuqua *et al*., 2001; Pappas *et al*., 2004). In Gram-positive bacteria, oligopeptides are usually detected by membrane-bound receptors for quorum sensing (Havarstein *et al*., 1995; Novick and Geisinger, 2008; Thoendel and Horswill, 2010). Examination of the phylogenetic relationship among all LuxI/LuxR systems leads to the conclusion that these quorum-sensing systems are ancient and thus cell–cell communication arose early in evolution (Gray and Garey, 2001; Lerat and Moran, 2004). The majority of *luxI/luxR* genes are located contiguously on the chromosome and therefore presumably retain their pairwise functional relationships through co-evolution as single cassettes (Gray and Garey, 2001; Lerat and Moran, 2004). The same genomic arrangement is observed for peptide-based quorum-sensing systems in Gram-positive bacteria (Pestova *et al*., 1996; Novick and Geisinger, 2008). That is, the gene encoding the autoinducer peptide is linked to the gene encoding the receptor. When we examine the gene organization of the different CqsA/CqsS homologues, we find that the *cqsA* and *cqsS* genes are usually adjacent in *Vibrio* species. In other species that possess homologous systems, such as *L. pneumophila*, *Burkholderia xenovorans* and *Ralstonia eutropha*, the genes that encode the synthase and the receptor are also contiguous (as in *R. eutropha*) or at least in close proximity with an occasional insertion of an accessory gene (e.g. in *L. pneumophila* and *B. xenovorans*). Finally, signal biosynthesis and signal detection profiles in *V. cholerae* and *V. harveyi* match closely (Figs 3 and 4), further suggesting that the *cqsA* and *cqsS* genes in each species coevolved. We therefore suggest that an ancestral CqsA/CqsS system must have diverged to give rise to the *V. cholerae* and *V. harveyi* CqsA/CqsS systems. *V. cholerae* and *V. harveyi* reside in different environmental niches. *V. cholerae* is a human pathogen that cycles between periods in the aquatic environment and periods inside the host; while *V. harveyi* is a marine bacterium that is pathogenic to many marine vertebrates and invertebrates. Although we do not know the forces that drove these two species to evolve different signalling specificities, we suspect that stringent signalling in the case of *V. harveyi* and promiscuous signalling in the case of *V. cholerae* increases the fitness of each species in its respective environmental niche.

## Experimental procedures

### Bacterial strains and culture conditions

All *V. cholerae* strains are derivatives of wild-type C6706str (Thelin and Taylor, 1996). All *V. harveyi* strains are derivatives of wild-type *V. harveyi* BB120 (Bassler *et al*., 1997). *E. coli* S17-1 p*ir*, DH5α and Top10 were used for cloning. Strains JMH626 and WN1102 were used in bioluminescence assays (see below). JMH626 is a *V. harveyi*Δ*cqsA*Δ*luxQ*Δ*luxN* mutant that does not produce Vh-CAI-1 due to the *cqsA* mutation. In addition, JMH626 does not detect HAI-1 and AI-2 because it lacks both the LuxN and LuxQ receptors (Henke and Bassler, 2004). WN1102 is a *V. cholerae*Δ*cqsA*Δ*luxQ* mutant that carries the *V. harveyi luxCDABE* operon on a cosmid. This strain does not produce CAI-1 and it does not detect AI-2. The relevant genotypes of all plasmids and strains are provided in *Supporting information*. Unless specified, *E. coli* was grown in LB medium at 37°C with shaking, *V. cholerae* and *V. harveyi* were grown in LM medium at 30°C with shaking. Unless specified, antibiotic concentrations are as follows: ampicillin, gentamicin and kanamycin, 100 mg l^−1^; chloramphenicol and tetracycline, 10 mg l^−1^; streptomycin, 5 g l^−1^; polymyxin B, 50 U l^−1^.

### DNA manipulation, site-directed mutagenesis and mutant construction

All DNA manipulations were performed using standard procedures (Sambrook *et al*., 1989). Oligonucleotide sequences used for PCR, site-directed mutagenesis and sequencing reactions will be provided upon request. Site-directed mutageneses were performed with QuikChange II XL Site-Directed Mutagenesis Kit according to the manufacturer's instructions. To examine the effects of *cqsS* mutations on the CAI-1 response of *V. cholerae*, point mutations were first constructed in pDH345 (Ng *et al*., 2010), and mutated *cqsS* alleles were subcloned into the suicide plasmid pKAS32 (Skorupski and Taylor, 1996). The mutated *cqsS* allele was introduced onto the *V. cholerae* chromosome using pKAS32 as previously described (Skorupski and Taylor, 1996). The pBB1 cosmid carrying the *V. harveyi luxCDABE* operon was introduced into *V. cholerae* strains via conjugation. To examine the effects of *cqsS* mutations on the CAI-1 response in *V. harveyi*, point mutations were first constructed in plasmid pJMH280 (Henke and Bassler, 2004), mutated *cqsS* alleles were subcloned into the low-copy-number cosmid pLAFR2, and the constructs were introduced into the *V. harveyi*Δ*cqsS* strain via conjugation, and the mutant *cqsS* alleles were maintained as episomes.

### Bioluminescence assay for *V. cholerae* and *V. harveyi* CqsS agonists

Culture fluids were prepared from overnight cultures unless otherwise specified. Cells were removed from the fluids by centrifugation and subsequent filtration through 0.22 µm filters. To assess CAI-1 activity in these cell-free fluids, overnight cultures of reporter strains were grown in LM medium (for *V. harveyi*) or LM with tetracycline (for *V. cholerae* carrying pBB1 and *V. harveyi* carrying *cqsS* on pLAFR2) and diluted 20-fold with sterile medium. Approximately 10–30% (v/v) of spent culture fluids were added to the diluted reporter strains. Bioluminescence and OD_600_ were measured in an Envison Multilabel Reader following 4 h incubation at 30°C with shaking. Sterile medium was used as the negative control. Synthetically prepared CAI-1 analogues were dissolved in DMSO and supplied at varying concentrations to the reporter strains. DMSO was used as the negative control.

### Extraction and purification of *V. harveyi* Ea-C8-CAI-1

*Escherichia coli* overexpressing *V. harveyi cqsA* (WN1327) was grown overnight in LB with kanamycin at 30°C with shaking. The culture was diluted 1000-fold in M9 minimal salts medium (Sigma) supplemented with 0.05% (w/v) leucine, 2 mM MgCl_2_, 0.1 mM CaCl_2_, 0.01% (w/v) thiamine and 0.4% (w/v) glucose with 10 mg l^−1^ kanamycin. The culture was incubated overnight at 30°C with shaking. Cells were removed by centrifugation and the cleared fluid was extracted by DCM. The extract was carefully concentrated without heating to a small volume (5 ml) in a Rotovap. The concentrated extract was fractionated using HPLC (see *Supporting information*). Each HPLC fraction was assayed in triplicate for *V. harveyi* CqsS agonist activity using the reporter strain JMH626 as described above. Fractions containing activity were further characterized (*Supporting information*).

### Purification of CqsA

The *V. cholerae* CqsA enzyme was purified as previously described (Kelly *et al*., 2009). For purification of *V. harveyi* CqsA, the open reading frame encoding *V. harveyi* CqsA was amplified by PCR and cloned into plasmid pET28B that had been previously digested with NdeI and BamHI. The resulting plasmid was transformed into *E. coli* BL21 Gold (DE3) resulting in strain WN1666. Strain WN1666 was grown in LB with kanamycin at 30°C with shaking until the OD_600_ of the culture reached ∼1.0. IPTG was added at a final concentration of 100 µM, and the culture was incubated for an additional 4 h at 30°C with shaking. Cells were harvested by centrifugation, suspended in lysis buffer (20 mM HEPES pH 8.0, 0.2 M NaCl, 5 mM β-mercaptoethanol, 100 µM pyridoxal-5′-phosphate, 1 mM MgCl_2_ and 20 mM imidazole), and lysed using a Cell Cracker. Soluble materials were loaded onto a HiTrap chelating column charged with nickel, the column was washed extensively with lysis buffer, and His_6_-tagged *V. harveyi* CqsA was eluted using a linear gradient of increasing concentration of imidazole dissolved in lysis buffer. Fractions containing CqsA were pooled, the buffer was exchanged, and protein was stored at −20°C in storage buffer [20 mM HEPES pH 8.0, 0.2 M NaCl, 1 mM DTT, 50% (v/v) glycerol]. Protein concentrations were determined by Bio-Rad Protein Assay Reagent.

### Coupled-enzyme assay for CqsA enzyme kinetics

A coupled-enzyme assay (modified from Hunter and Ferreira, 1995; Webster *et al*., 2000) was used to measure the rate of CoA production by CqsA in the presence of SAM and different acyl CoA substrates. Released CoA is reacted with α-ketoglutarate and NAD^+^ to form succinyl CoA, NADH and CO_2_ when excess α-ketoglutarate dehydrogenase is supplied. The rate of NADH production is followed at 340 nm using a spectrophotometer. Briefly, the rates of CoA release from *V. cholerae* and *V. harveyi* CqsA enzymes were measured in reactions containing 20 mM potassium phosphate buffer pH 7.0, 3 mM MgCl_2_, 1 mM NAD^+^, 1 mM α-ketoglutarate, 200 µM SAM, 1 unit of α-ketoglutarate dehydrogenase (Sigma) and 60–300 nM CqsA. Decanoyl CoA or octanoyl CoA was added to the reactions at concentrations of 1–200 µM. The rate of CoA production was monitored for 30 min. Data were fitted using Graphpad Prism to obtain the kinetic parameters.

### Measurement of the concentration of CAI-1 type molecules in *V. harveyi*, *V. cholerae* and other *Vibrio* species culture fluids

Bacteria were grown in LM medium at 30°C overnight with shaking. The cultures were diluted 200-fold in M9 minimal salts medium (Sigma) supplemented with 2 mM MgCl_2_, 0.1 mM CaCl_2_, 1× MEM vitamins (Sigma), 0.3 M NaCl, 0.5% (w/v) glucose and incubated at 30°C with shaking. Cell growth was monitored at OD_600_. When OD_600_ reached ∼1.0, cells were removed by centrifugation. Pure d_2_-CAI-1 was added to 9 ml of collected cell-free fluids at 500 nM as an internal control. The mixtures were extracted into 500 µl of DCM. The organic layer was separated and 100 µl of this extract was diluted into 100 µl of HRMS solvent (9:1 acetonitrile : H_2_O with 0.1% formic acid), and this sample was injected directly for HRMS analysis. Due to variability in the aqueous/organic partitioning during extraction and the different ionization potentials of the CAI-1 type molecules, we determined a correction factor for each molecule by preparing a mixed sample containing known concentrations of each of the CAI-1 type molecules together with an internal standard (d_2_-CAI-1) in M9 medium. This sample was treated in an identical manner to the cell-free fluids described above.

### Chemical synthesis and analytical methods

All chemical syntheses and analytical methods are provided in *Supporting information*.
