# Supplementary material for: Signal production and detection specificity in Vibrio CqsA/CqsS quorum-sensing systems
Source: Mol Microbiol. 2011 Jan 26;79(6):1407–17. doi: 10.1111/j.1365-2958.2011.07548.x (PMC3285556; doi:10.1111/j.1365-2958.2011.07548.x)
Supplement: Supplementary file 1 [file mmi0079-1407-SD1.pdf]

**SUPPORTING INFORMATION**

1. **Strains and plasmids table**

2. **HPLC and SFC conditions**

3. **HRMS analyses (raw data and corrective factors)**

4. **Synthesis of Ea-C8-CAI-1 by *V. harveyi* CqsA in vitro and in vivo**

5. **Chemical synthesis**

6. **NMR analyses of natural and synthetic CAI-1 type compounds**

**Supplementary table 1. Strains and plasmids**

| Plasmid or Strain | Relevant genotype or feature | Reference |
|---|---|---|
| **Plasmid** | | |
| pKAS32 | Suicide vector for allelic exchange in *V. cholerae,* $Amp^R$ | (Skorupski and Taylor, 1996) |
| pLAFR2 | Broad-host-range cosmid; *mob*, $Tet^R$ | (Friedman *et al.*, 1982) |
| pBB1 | *V. harveyi luxCDABE* on pLAFR2, $Tet^R$ | (Miller *et al.*, 2002) |
| pDH345 | *V. cholerae cqsS* on pSLS4, $Kan^R$ | (Ng *et al.*, 2010) |
| pWN1365 | *V. cholerae* $cqsS^{C170F}$ on pDH345, $Kan^R$ | This study |
| pWN1960 | *V. cholerae* $cqsS^{C170F}$ on pKAS32, $Amp^R$ | This study |
| pJMH280 | *V. harveyi cqsS* on pGEM-T, $Amp^R$ | (Henke and Bassler, 2004) |
| pWN1513 | *V. harveyi* $cqsS^{F175C}$ on pJMH280, $Amp^R$ | This study |
| pJMH282 | *V. harveyi cqsS* on pLAFR2, $Tet^R$ | (Henke and Bassler, 2004) |
| pWN1515 | *V. harveyi* $cqsS^{F175C}$ on pLAFR2, $Tet^R$ | This study |
| pWN1327 | *V. harveyi cqsA* on pET-28B, untagged, $Kan^R$ | This study |
| WN1666 | *V. harveyi cqsA* on pET-28B, N-terminal $His_6$-tagged, $Kan^R$ | This study |
| ***Vibrio cholerae*** | | |
| C6706str | Wild type | (Thelin and Taylor, 1996) |
| BH1523 | Δ*cqsA* | This study |
| DH197 | Δ*cqsA,* Δ*luxQ* | This study |

| | | |
|---|---|---|
| WN1102 | Δ*cqsA*, Δ*luxQ*, pBB1 | This study |
| WN1981 | Δ*cqsA*, Δ*luxQ*, *cqsS*$^{C170Y}$ | This study |
| WN1993 | Δ*cqsA*, Δ*luxQ*, *cqsS*$^{C170Y}$/pBB1 | This study |
| ***Vibrio harveyi*** | | |
| BB120 | Wild type | (Bassler *et al.*, 1997) |
| JMH603 | Δ*cqsA* | (Henke and Bassler, 2004) |
| JMH626 | Δ*cqsA*, Δ*luxN*, Δ*luxQ* | (Henke and Bassler, 2004) |
| WN1397 | Δ*cqsA*, Δ*cqsS*, Δ*luxN*, Δ*luxPQ* | This study |
| WN1492 | Δ*cqsA*, Δ*cqsS*, Δ*luxN*, Δ*luxPQ*/pJMH282 | This study |
| WN1834 | Δ*cqsA*, Δ*cqsS*, Δ*luxN*, Δ*luxPQ*/pWN1515 | This study |

## 2. HPLC and SFC conditions

For NP-HPLC, four liters of the cell-free supernant from an M9 culture *E. coli* overexpressing *V. harveyi* CqsA (see Experimental Procedures for details) was extracted into MTBE (4 x 500 mL), the combined organic extract was washed with a saturated brine solution (1 L) and was dried over $Na_2SO_4$. The dried organics were carefully concentrated *in vacuo* at room temperature to yield a concentrated organic extract in ~2-5 mL of organic solvent. Complete concentration of this sample leads to significant loss of biological activity. This concentrated organic sample was diluted with hexanes (5 mL) and was purified by preperative HPLC on a system composed 2 PrepStar SD-1 pumps, a Knauer K-2501 multi-wavelength detector set at 280nm, a Rainin FC-1 fraction collector and using a PrincetonSPHER Premier (2 x 25 cm) column. The mixture was fractionated by application of a gradient from 9:1 hexane:MTBE to 100% MTBE at 20 mL/min over 25 min, with fractions collected every minute. Ea-C8-CAI-1 (by HRMS and bioassay) has a retention time of 8 minutes, C8-CAI-1 and the α-hydroxy ketone regioisomer (by HRMS, NMR and bioassay) elute as two smaller peaks between 9 and 10 minutes.

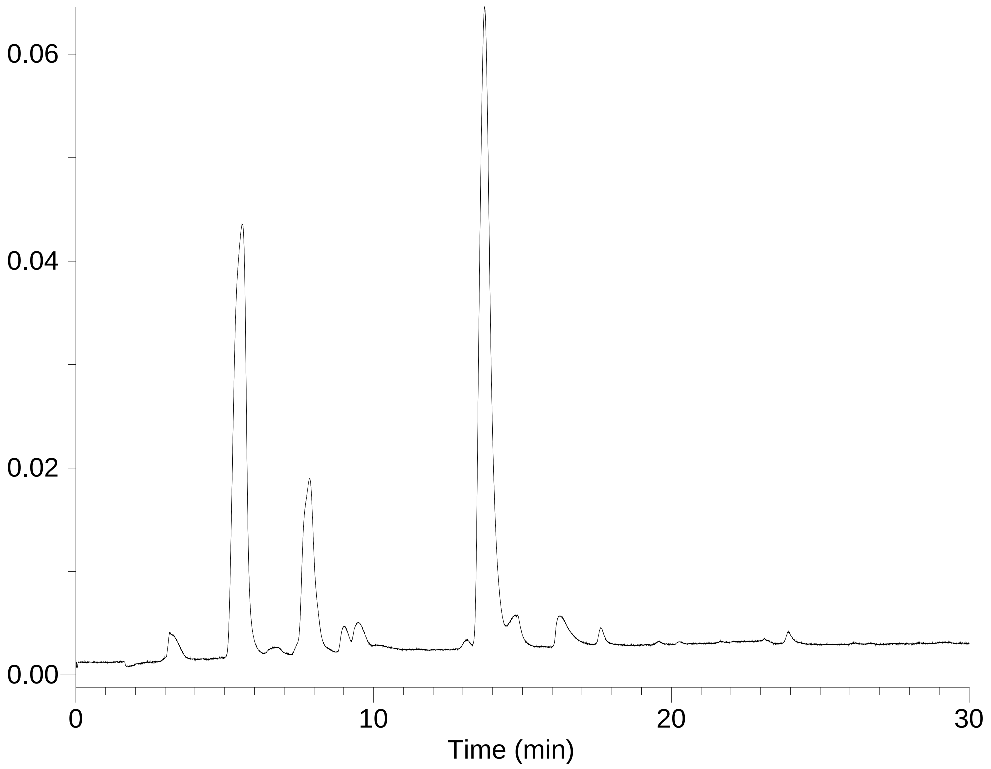

Due to the observed instability of purified or unpurified Ea-C8-CAI-1, we turned to an alternate strategy for purification. Accordingly, we developed a SFC (supercritical fluid chromatography) method using liquid $CO_2$ along with a small amount of per-deuterated methanol (d4-MeOH) as a co-solvent. Importantly, this approach directly provides concentrated fractions in d4-MeOH, suitable for direct NMR analysis.

For the purification, four liters of the cell-free supernant from an M9 culture *E. coli* overexpressing *V. harveyi* CqsA (see Experimental Procedures for details) was extracted into MTBE (4 x 500 mL), the combined organic extract was washed with a saturated brine solution (1 L) and was dried over $Na_2SO_4$. The dried organics were carefully concentrated *in vacuo* at room temperature to yield a concentrated organic extract in ~2-5 mL of organic solvent. Purification was achieved using a Berger Multigram II SFC system equipped with 2 Varian SD-1 pumps, a Knauer K-2501 multi-wavlength dectector set at 280nm, a Knauer K-1900 pump, a Vatran SGP-50-100 condenser, using a PrincetonSPHER Premier (2 x 25 cm) column. Fractionation of the crude mixture was achieved by application of an isocratic method using a mixture of 8% d4-MeOH/$CO_2$ (100 bar) at 50 mL/min. Fractions were collected manually and the product was found to have a retention time of 3.5 min. This fraction was subjected to direct $^1$H-NMR analysis, and found to contain a mixture of Ea-C8-CAI-1 along with C8-CAI-1 and its α-hydroxy ketone regioisomer.

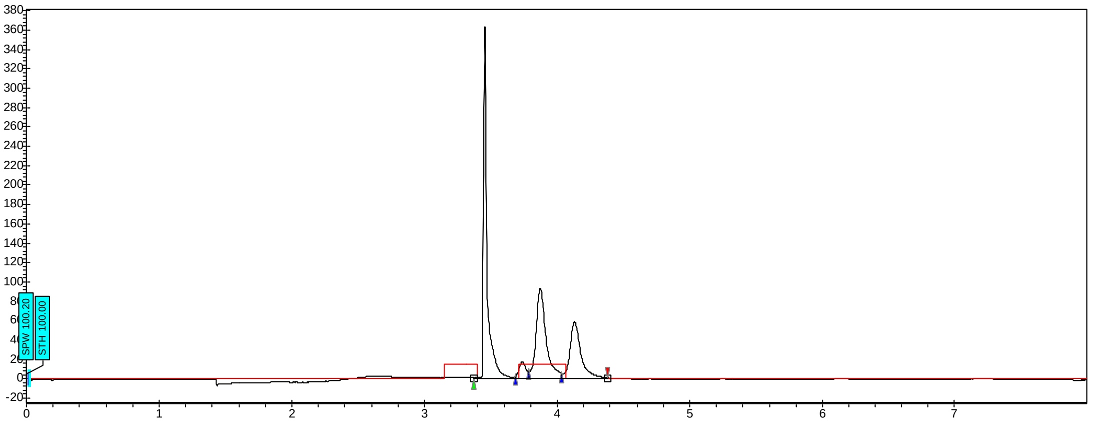

### 3. HRMS analyses (Corrective factor determination and cell-free fluids analyses)

A. HRMS data for corrective factor determination:

| Molecule | ion abundance | | | multiplicative factor of molecular ion to $d_2$-CAI1 | | | | |
|---|---|---|---|---|---|---|---|---|
| | Sample 1 | Sample 2 | Sample 3 | Sample 1 | Sample 2 | Sample 3 | Average | Standard Deviation |
| Ea-C8-CAI-1 | 49.4 | 183.7 | 263 | 1.30 | 0.81 | 0.74 | 0.95 | 0.31 |
| C8-CAI-1 | 57.6 | 174.1 | 192.2 | 1.12 | 0.85 | 1.02 | 1.00 | 0.14 |
| Ea-CAI-1 | 33 | 73.5 | 108.9 | 1.95 | 2.01 | 1.80 | 1.92 | 0.11 |
| $d_2$-CAI-1 | 64.4 | 148.1 | 195.6 | 1.00 | 1.00 | 1.00 | 1.00 | 0.00 |

B. HRMS data for the *V. harveyi* cell-free fluids:

| Molecule | Ion Abundance from HRMS | | | Calculated Concentration (nM) | | | **Average Concentration (nM)** | **Standard Deviation** |
|---|---|---|---|---|---|---|---|---|
| | Sample 1 | Sample 2 | Sample 3 | Sample 1 | Sample 2 | Sample 3 | | |
| Ea-C8-CAI-1 | 203.5 | 329.6 | 135.7 | 62.3 | 49.5 | 36.6 | 49.5 | 12.8 |
| C8-CAI-1 | 62 | 104.6 | 34.2 | 198.6 | 165.0 | 97.0 | 153.5 | 51.8 |
| Ea-CAI-1 | 0 | 0 | 0 | 0.0 | 0.0 | 0.0 | 0.0 | 0.0 |
| CAI-1 | 0 | 0 | 0 | 0.0 | 0.0 | 0.0 | 0.0 | 0.0 |
| $d_2$-CAI-1 | 155.4 | 316.9 | 176.3 | 500.0 | 500.0 | 500.0 | 500.0 | 0.0 |

C. HRMS data for the *V. cholerae* cell-free fluids:

| Molecule | Ion Abundance from HRMS | | | Calculated Concentration (nM) | | | **Average concentration (nM)** | **Standard Deviation** |
|---|---|---|---|---|---|---|---|---|
| | Sample 1 | Sample 2 | Sample 3 | Sample 1 | Sample 2 | Sample 3 | | |
| Ea-C8-CAI-1 | 141.3 | 83.9 | 92.1 | 23.1 | 15.0 | 19.8 | 19.3 | 4.1 |
| C8-CAI-1 | 0 | 0 | 0 | 0.0 | 0.0 | 0.0 | 0.0 | 0.0 |
| Ea-CAI-1 | 348.7 | 321.1 | 320.5 | 115.2 | 116.0 | 139.0 | 123.4 | 13.5 |
| CAI-1 | 132.3 | 111.6 | 98.2 | 227.6 | 209.9 | 221.8 | 219.8 | 9.0 |
| $d_2$-CAI-1 | 290.7 | 265.8 | 221.4 | 500.0 | 500.0 | 500.0 | 500.0 | 0.0 |

The detection limits for the above molecules were determined to be: Ea-C8-CAI-1, 6.25 nM; C8-CAI-1, 25 nM; Ea-CAI-1, 12.5 nM; CAI-1, 25 nM. This was determined by the analysis of a series of samples containing pure samples of each of the listed molecules at concentrations of 50 nM, 25 nM, 12.5 nM, 6.25 nM, 3.125 nM, and 1.56 nM. Detection limits are described as the last sample for which a HRMS peak was observed.

**4. Ea-C8-CAI-1 synthesis by *V. harveyi* CqsA *in vitro* (A) and *in vivo* (B)**

(A) The substrate required for *V. harveyi* CqsA to produce Ea-C8-CAI-1 was tested in buffer (10 mM HEPES, 0.1 M NaCl) in the presence or absence of C8-CoA (100 μM), SAM (1 mM), and *V. harveyi* CqsA (500 nM). The reaction mixture was incubated at room temperature for 1 hr and terminated by addition of an equal volume of acetonitrile. The reaction mixture was assayed for stimulation of bioluminescence expression in the *V. harveyi* CAI-1 reporter strain JMH626. Only reactions containing CqsA, SAM, and C8 CoA resulted in induction of bioluminescence. HRMS analysis of the reaction products revealed the molecular ion of Ea-C8-CAI-1. RLU denotes relative light units.

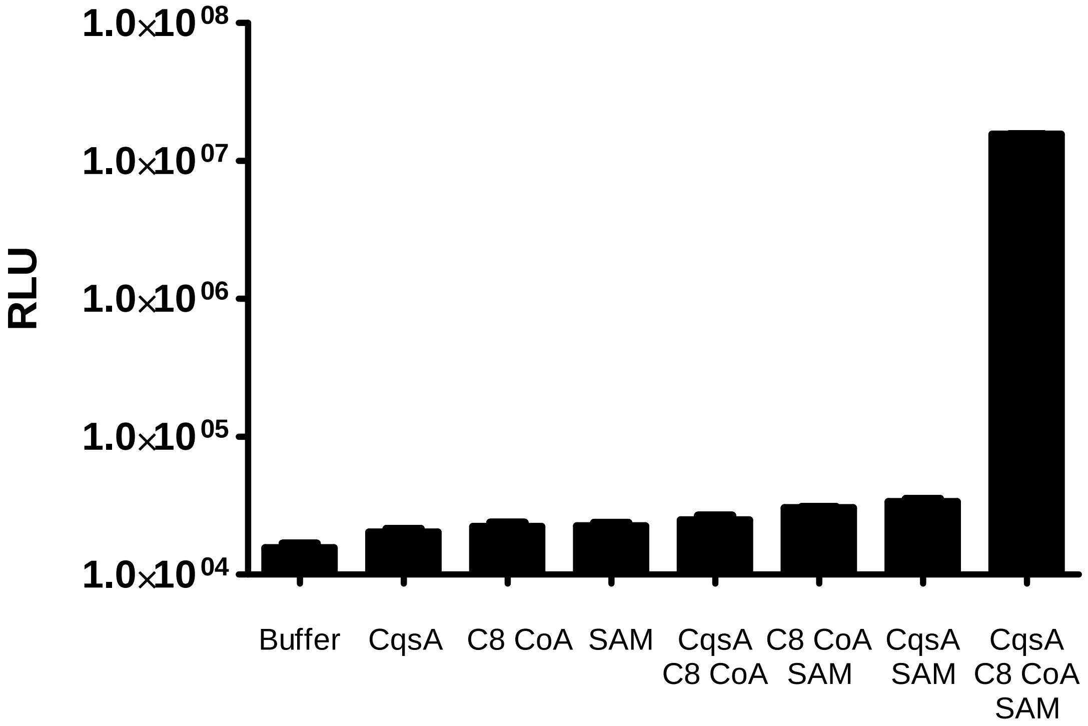

(B) Heavy atom labeling was used to examine whether SAM could be used *in vivo* by CqsA as a substrate. *E. coli* overexpressing *V. harveyi* CqsA (WN1327, top) produced Ea-C8-CAI-1 (184.169 ion) and C8-CAI-1 (187.169 ion). A *metE E. coli* mutant (WN1590, middle) grown in the presence of unlabeled methionine carrying the CqsA overexpression plasmid produced the identical two molecules. Consistent with the idea that SAM is used *in vivo* for Ea-C8-CAI-1 synthesis and C8-CAI-1 is derived from Ea-C8-CAI-1, WN1590 grown in the presence of d8-L-methionine (bottom) produced triple-deuterium labeled Ea-C8-CAI-1 (187.189 ion) and C8-CAI-1 (190.188 ion) (See (Wei *et al.*, 2010) for detailed explanations).

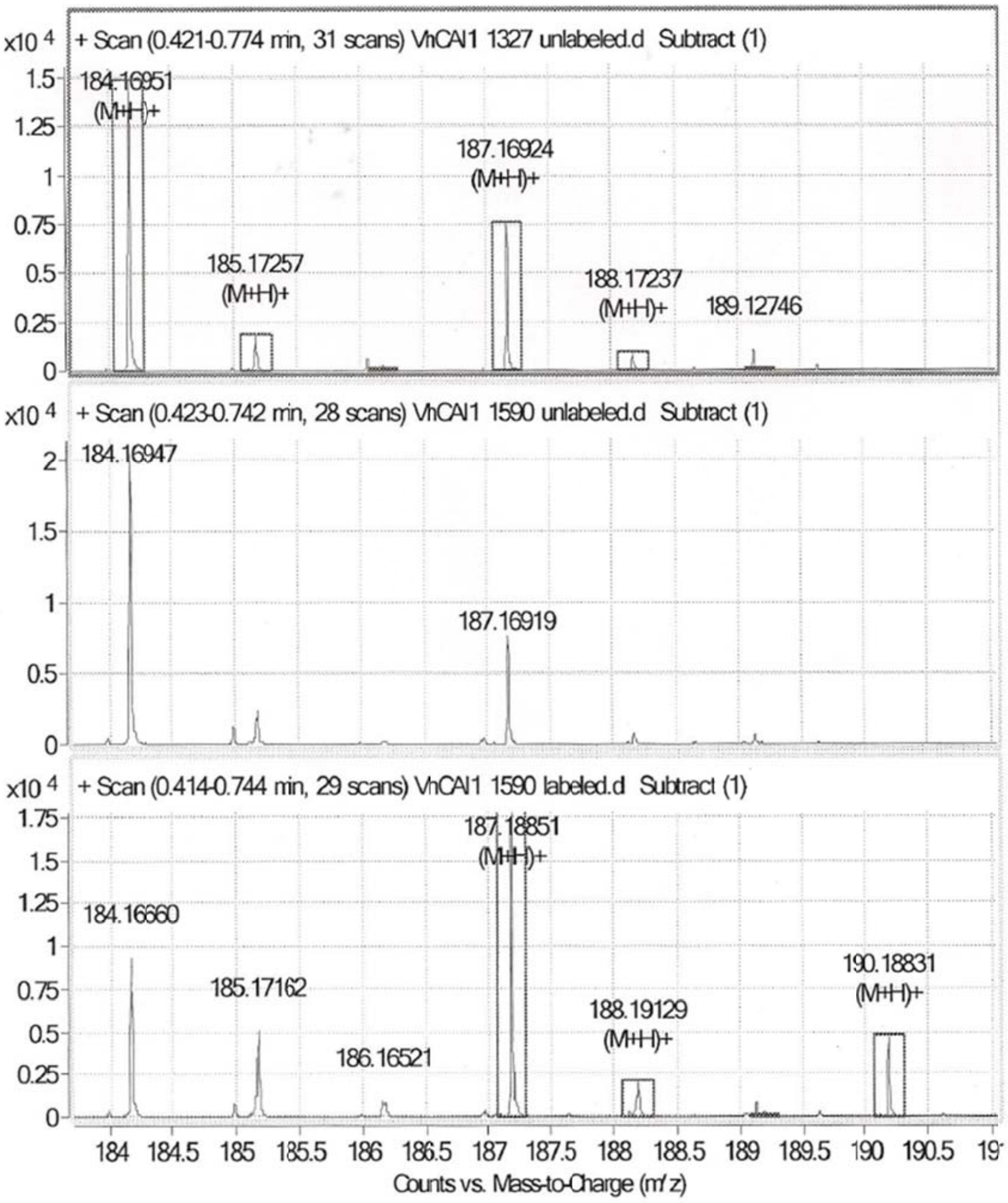

**5. Chemical Syntheses.**

**Analytical methods.** NMR spectra were recorded using a Bruker Avance II spectrometer (500 MHz for $^{1}H$; 125 MHz for $^{13}C$) equipped with either a $^{1}H$-optimized TCI (H/C/N) cryoprobe or a $^{13}C$-optimized dual C/H cryoprobe. Chemical shifts are reported in parts per million (ppm) and were calibrated according to residual solvent. High-resolution mass spectral analyses were performed using an Agilent 1200-series electrospray ionization–time-of-flight (ESI-TOF) mass spectrometer in the positive ESI mode. Note, the NMR data for synthetic and natural Ea-CAI-1 also appear in the supplement to Wei *et al*., 2010 as evidence for production of Ea-CAI-1 and conversion to CAI-1 in *V. cholerae*.

**Chemical Reactions.** Unless otherwise noted, all reactions were performed in flame-dried glassware under an atmosphere of nitrogen. All chemicals purchased from commercial vendors were used without further purification. Anhydrous Sure/Seal™ solvents were purchased from commercial vendors.

**Purification.** Flash chromatography was performed using standard grade silica gel 60 230-400 mesh from SORBENT Technologies. Analytical thin-layer chromatography was carried out using Silica G TLC plates, 200 µm with $UV_{254}$ fluorescent indicator (SORBENT Technologies), and visualization was performed by staining (anisaldehyde, ceric ammonium molybdate, or ninhydrin) and/or by absorbance of UV light.

All synthetic molecules were determined to be pure by NMR and HRMS, Ea-CAI-1 and C8-Ea-CAI-1 were not purified and were characterized directly from the final deprotection step. NMR data of these products containing peaks consistent with the removed protecting group (TIPS-F) and internal standard (PhMe) are provided.

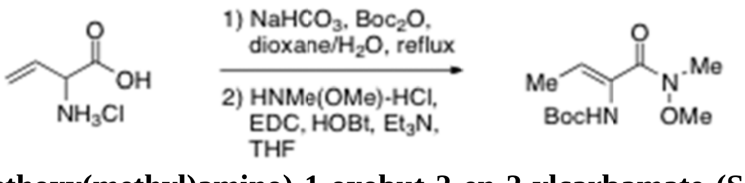


**(*Z*)-*tert*-butyl 1-(methoxy(methyl)amino)-1-oxobut-2-en-2-ylcarbamate (S1).** L-Vinylglycine hydrochloride (Afzali-Ardakani and Rapoport, 1980; Carrasco *et al.*, 1992)(2.14 g, 15.6 mmol) was dissolved in dioxane/$H_2O$ (182 mL, 0.085 M, 1:1 dioxane:$H_2O$) and was treated with

$NaHCO_3$ (2.62 g, 31.1 mmol, 2.0 eq) and $Boc_2O$ (3.56 g, 16.3 mmol, 1.05 eq) sequentially at room temperature. The resulting mixture was heated to reflux for 2.5 hr, was cooled and concentrated *in vacuo* to a volume of ca. 80 mL. The resulting solution was acidified with 1 N HCl to ~pH 6.5, was extracted with DCM (3 x 50 mL), dried over $Na_2SO_4$ and concentrated to dryness. The resulting oil (2.82 g) was dissolved in THF (156 mL, 0.1 M to VGly-HCl) at ambient temperature and was treated with HOBt (6.3 g, 46.7 mmol, 3.0 eq to VGly-HCl), HNMe(OMe)-HCl (1.75 g, 17.9 mmol, 1.15 eq to VGly-HCl), EDC (3.43 g, 17.9 mmol, 1.15 eq to VGly-HCl), and $Et_3N$ (10.8 mL, 77.8 mmol, 5.0 eq to VGly-HCl) sequentially and the mixture was allowed to stir for 8 hr at ambient temperature. The crude mixture was filtered through a plug of celite and concentrated *in vacuo*. The resulting oil was purified by silica gel chromatography eluting with a gradient from hexanes to 60% EtOAc/hexanes. Fractions containing the desired product were combined and concentrated *in vacuo* to provide **S1** as a pale yellow oil (2.2 g, 58% from VGly-HCl). $^1$H-NMR (500 MHz, $CDCl_3$) δ 6.22 (bs, 1H), 5.67 (bs, 1H), 3.64 (s, 3H), 3.22 (s, 3H), 1.68 (d, *J* = 7.0 Hz, 3H), 1.41 (s, 9H); $^{13}$C-NMR (125 MHz, $CDCl_3$) δ 167.4, 152.9, 129.5, 120.9, 80.5, 61.1, 34.4, 28.3, 12.6; HRMS (ESI-TOF) calcd for $C_{11}H_{20}N_2O_4Na$, 267.1321 *m*/*z* [M+Na]; observed, 267.1323 *m*/*z* $[M+Na]^+$. The configuration of this molecule was assigned on the basis of its $^1$H-NMR spectra [10].

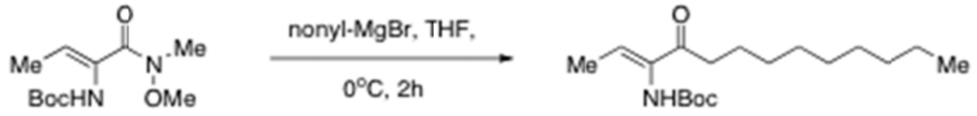


**(*Z*)-*tert*-butyl 4-oxotridec-2-en-3-ylcarbamate (S2).** To a solution of (*Z*)-*tert*-butyl 1-(methoxy(methyl)amino)-1-oxobut-2-en-2-ylcarbamate (**S1**) (172.5 mg, 0.71 mmol) in THF (7 mL, 0.1 M) at 0°C was added nonyl-MgBr (3.5 mL, 3.5 mmol, 5.0 eq, 1.0 M in diethyl ether) and the mixture was stirred at 0°C for 2 h. The resulting mixture was quenched with sat. $NH_4Cl$ (20 mL), extracted with $Et_2O$ (3 x 20 mL), combined organics were washed with sat. NaCl (20 mL), dried over $Na_2SO_4$ and concentrated *in vacuo*. The resulting oil was purified by silica gel chromatography eluting with a gradient from hexanes to 20% EtOAc/hexanes to provide **S2** as a clear colorless oil (166.3 mg, 76%). $^1$H-NMR (500 MHz, $CDCl_3$) δ 6.50 (q, *J* = 7.1 Hz, 1H), 6.42 (s, 1H), 2.64 (t, *J* = 7.5 Hz, 2H), 1.85 (d, *J* = 7.1 Hz, 3H), 1.65-1.55 (m, 2H), 1.44 (s, 9H), 1.32-

1.16 (m, 12H), 0.86 (t, $J$ = 6.9 Hz, 3H); $^{13}$C-NMR (125 MHz, $CDCl_3$) δ 198.0, 153.1, 135.3, 130.4, 80.5, 36.9, 32.1, 29.7, 29.6, 29.5, 29.5, 28.4, 24.9, 22.9, 15.3, 14.3; HRMS (ESI-TOF) calcd for $C_{18}H_{33}NO_3Na$ 334.2358 *m*/*z* [M+Na]; observed, 334.2351 *m*/*z* $[M+Na]^+$.

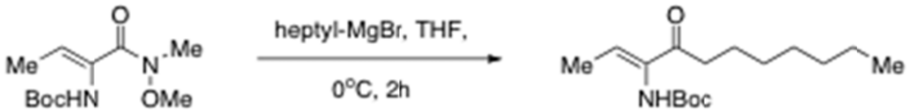


**(*Z*)-*tert*-butyl 4-oxoundec-2-en-3-ylcarbamate (S3).** Prepared in an analogous manner to **S2** from (*Z*)-*tert*-butyl 1-(methoxy(methyl)amino)-1-oxobut-2-en-2-ylcarbamate (**S1**) and heptyl-MgBr in 71% yield. $^1$H-NMR (500 MHz, $CDCl_3$) δ 6.50 (q, $J$ = 7.1 Hz, 1H), 6.42 (s, 1H), 2.64 (t, $J$ = 7.5 Hz, 2H), 1.84 (d, $J$ = 7.1 Hz, 3H), 1.64-1.51 (m, 2H), 1.44 (s, 9H), 1.33-1.18 (m, 8H), 0.85 (t, $J$ = 6.9 Hz); $^{13}$C-NMR (125 MHz, $CDCl_3$) δ 198.0, 153.1, 135.3, 130.4, 80.5, 36.9, 31.9, 29.5, 29.3, 28.4, 24.9, 22.8, 15.3, 14.3; HRMS (ESI-TOF) calcd for $C_{16}H_{29}NO_3Na$ 306.2045 *m*/*z* [M+Na]; observed, 306.2041 *m*/*z* $[M+Na]^+$.

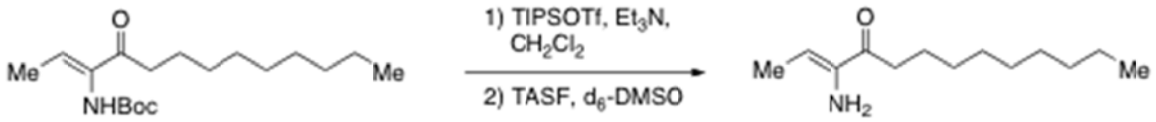


**(*Z*)-3-aminotridec-2-en-4-one (Ea-CAI-1).** To a solution of (*Z*)-*tert*-butyl 4-oxotridec-2-en-3-ylcarbamate (**S2**) (54.9 mg, 0.18 mmol) in $CH_2Cl_2$ (1.2 mL, 0.15 M) at 0°C was added $Et_3N$ (74 μL, 0.53 mmol, 3.0 eq) followed by TIPSOTf (142 μL, 0.53 mmol, 3.0 eq) and the mixture was stirred at 0°C for 2 h. To this mixture was added additional $Et_3N$ (74 μL, 0.53 mmol, 3.0 eq) and TIPSOTf (142 μL, 0.53 mmol, 3.0 eq) and the mixture was allowed to slowly warm to ambient temperature and was stirred for an additional 18 h. The prolonged reaction time, temperature profile, and the portionwise addition of $Et_3N$ and TIPSOTf were found to be important for optimal conversion. At this time, TLC analysis showed complete consumption of the starting material (Rf=0.2, 10% EtOAc/hexanes, ninhydrin) to a single new product spot (Rf=0.4, 10% EtOAc/hexanes, ninhydrin) and the reaction was quenched with sat. $NH_4Cl$ (10 mL), extracted with $CH_2Cl_2$ (3 x 10 mL), the combined organics were washed with sat. NaCl, dried over $Na_2SO_4$ and concentrated *in vacuo*. The resulting oil was flashed through a plug of silica gel,

eluting with 10% EtOAc/hexanes, was concentrated to dryness and used without further purification. The resulting oil was dissolved in d6-DMSO (2.1 mL with 3 μL PhMe as an internal standard), was treated with TASF (145.4 mg, 0.53 mmol, 3 eq to **S2**) and was vortexed for 5 min before NMR analysis. $^{1}$H-NMR analysis enabled determination of the concentration of Ea-CAI-1 by integration of the Ea-CAI-1 vinyl methyl doublet (δ 1.65) to the PhMe methyl singlet (δ 2.29). The concentration of Ea-CAI-1 determined in this manner was 13.6μM (0.029 mmol, 16% from **S2**). $^{1}$H-NMR (500 MHz, d6-DMSO) δ 5.53 (q, *J* = 7.1 Hz, 1H), 4.31 (s, 2H), 2.60 (t, *J* = 7.3 Hz, 2H), 1.65 (d, *J* = 7.0 Hz, 3H), 1.53-1.41 (m, 2H), 1.30-1.17 (m, 12H), 0.85 (t, *J* = 6.4 Hz, 3H); $^{13}$C-NMR (125 MHz, d6-DMSO) δ 197.1, 141.8, 106.9, 35.2, 31.4, 29.0, 29.0, 28.8, 25.1, 22.2, 14.0, 12.2; HRMS (ESI-TOF) calcd for $C_{13}H_{26}NO$ 212.2014 *m/z* [M+H]; observed, 212.2010 *m/z* $[M+H]^{+}$.

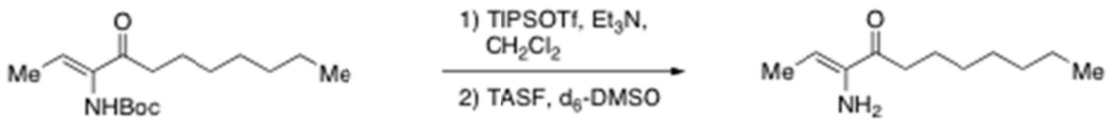


**(*Z*)-3-aminoundec-2-en-4-one (C8-Ea-CAI-1).** Prepared in an analogous manner to Ea-CAI-1 from (*Z*)-*tert*-butyl 4-oxoundec-2-en-3-ylcarbamate (**S3**) in 13% overall yield. $^{1}$H-NMR (500 MHz, d6-DMSO/PhMe internal standard) δ 5.51 (q, *J* = 7.1 Hz, 1H), 4.30 (s, 2H), 2.59 (t, *J* = 7.4 Hz, 2H), 1.65 (d, *J* = 7.1 Hz, 3H), 1.51-1.43 (m, 2H), 1.25-1.19 (m, 8H), 0.84 (t, *J* = 6.8 Hz, 3H); HRMS (ESI-TOF) calcd for $C_{11}H_{22}NO$ 184.1701 *m/z* [M+H]; observed, 184.1699 *m/z* $[M+H]^{+}$.

Refer to (Higgins *et al.*, 2007; Kelly *et al.*, 2009; Ng *et al.*, 2010) for syntheses of CAI-1, Am-CAI-1, C8-CAI-1, and Am-C8-CAI-1.

**6. NMR analyses of natural and synthetic CAI-1 type compounds.**

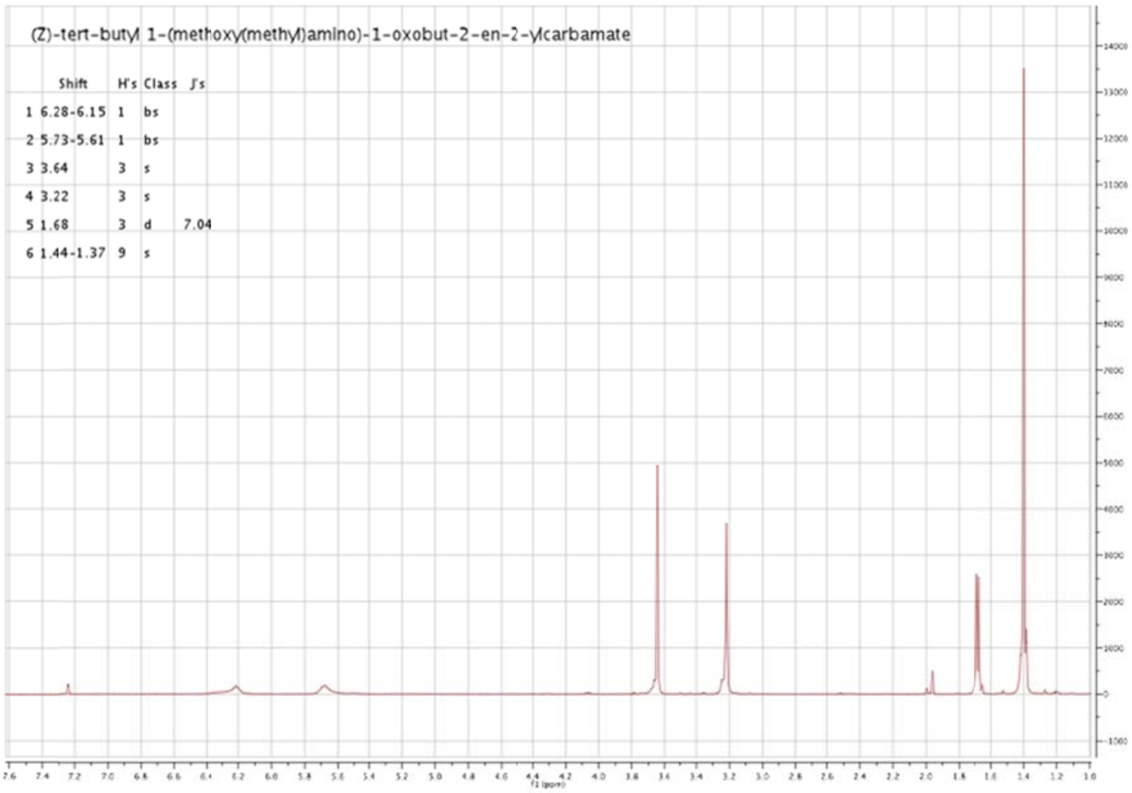


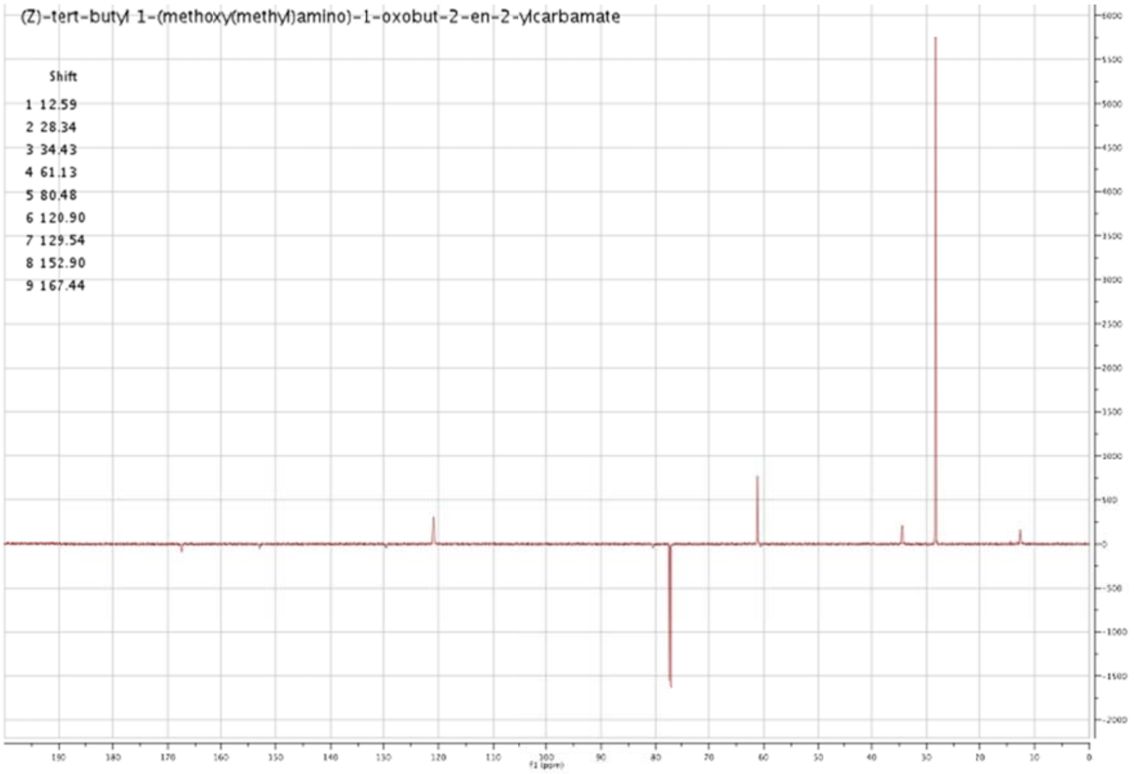

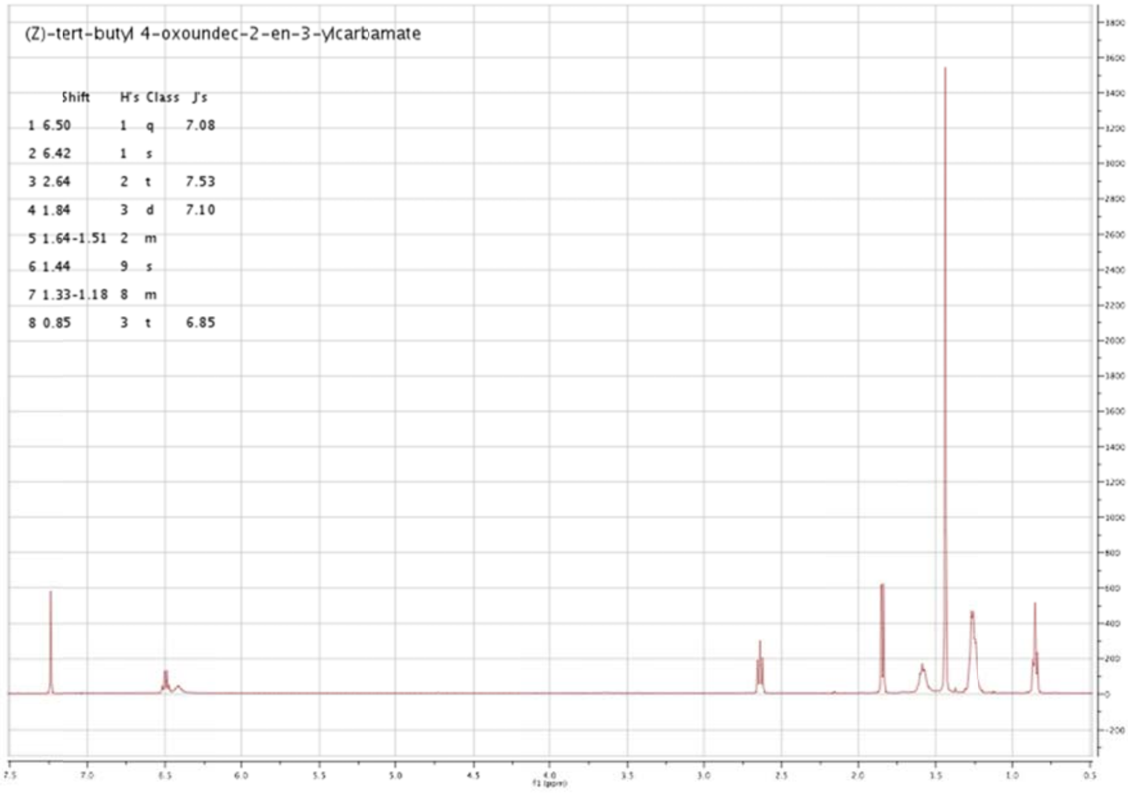
(Z)-tert-butyl 4-oxoundec-2-en-3-ylcarbamate
Shift H's Class J's
1 6.50 1 q 7.08
2 6.42 1 s
3 2.64 2 t 7.53
4 1.84 3 d 7.10
5 1.64-1.51 2 m
6 1.44 9 s
7 1.33-1.18 8 m
8 0.85 3 t 6.85
f1 (ppm)

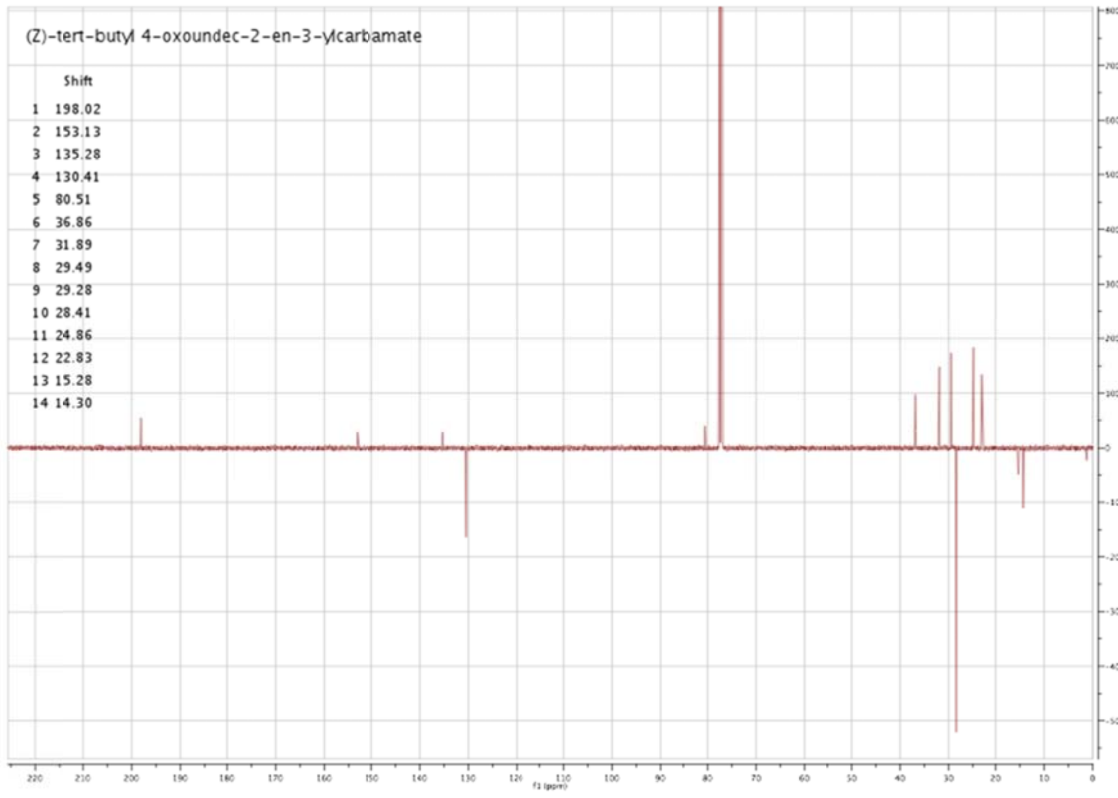
(Z)-tert-butyl 4-oxoundec-2-en-3-ylcarbamate
Shift
1 198.02
2 153.13
3 135.28
4 130.41
5 80.51
6 36.86
7 31.89
8 29.49
9 29.28
10 28.41
11 24.86
12 22.83
13 15.28
14 14.30
f1 (ppm)

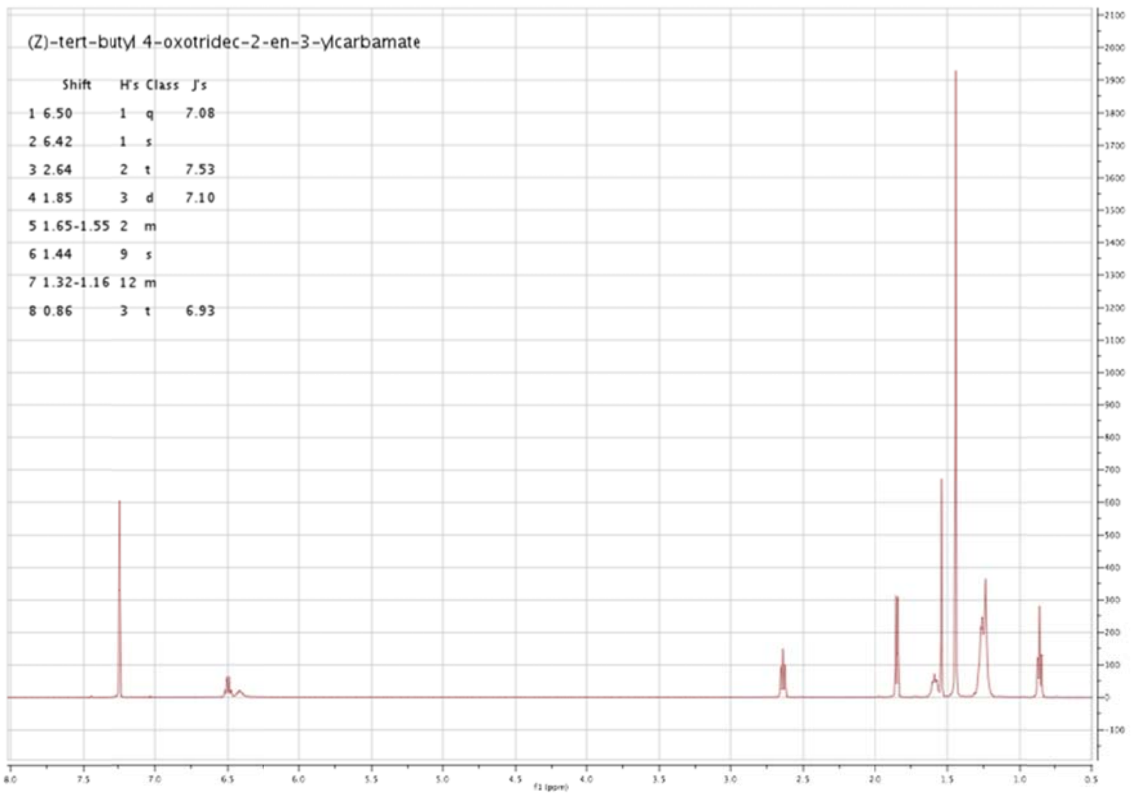
(Z)-tert-butyl 4-oxotridec-2-en-3-ylcarbamate
Shift H's Class J's
1 6.50 1 q 7.08
2 6.42 1 s
3 2.64 2 t 7.53
4 1.85 3 d 7.10
5 1.65-1.55 2 m
6 1.44 9 s
7 1.32-1.16 12 m
8 0.86 3 t 6.93
f1 (ppm)

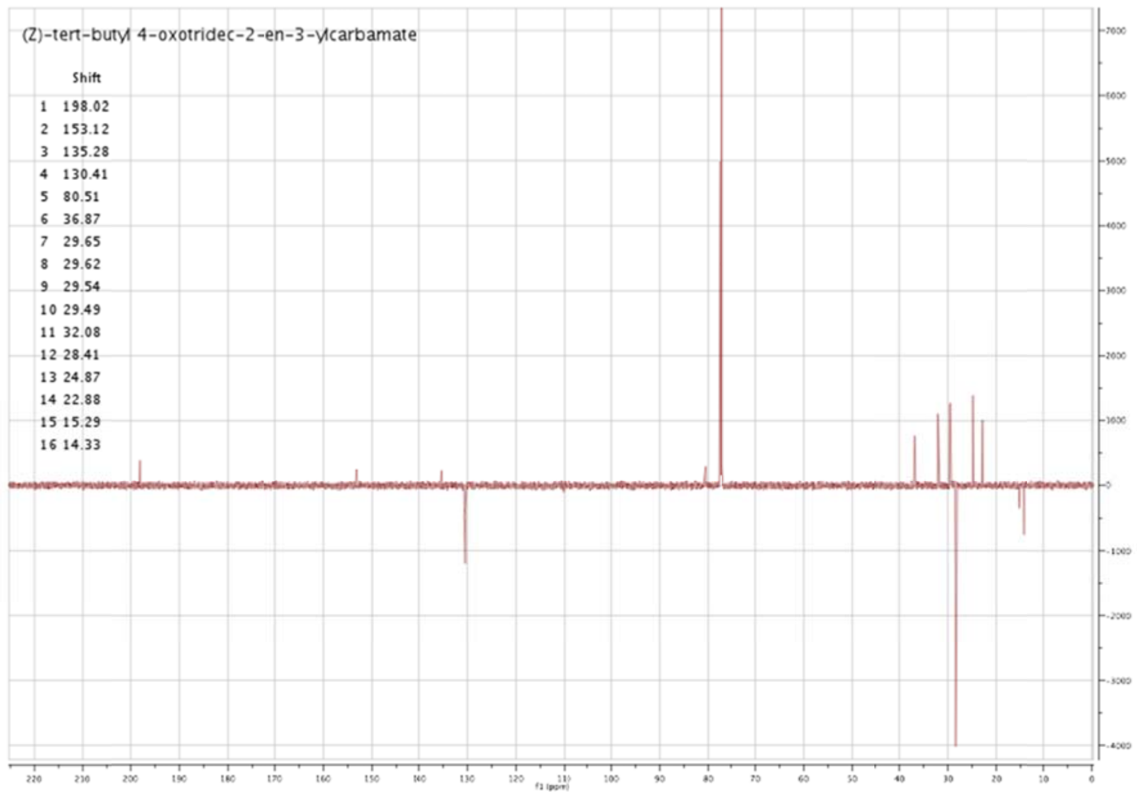
(Z)-tert-butyl 4-oxotridec-2-en-3-ylcarbamate
Shift
1 198.02
2 153.12
3 135.28
4 130.41
5 80.51
6 36.87
7 29.65
8 29.62
9 29.54
10 29.49
11 32.08
12 28.41
13 24.87
14 22.88
15 15.29
16 14.33
f1 (ppm)

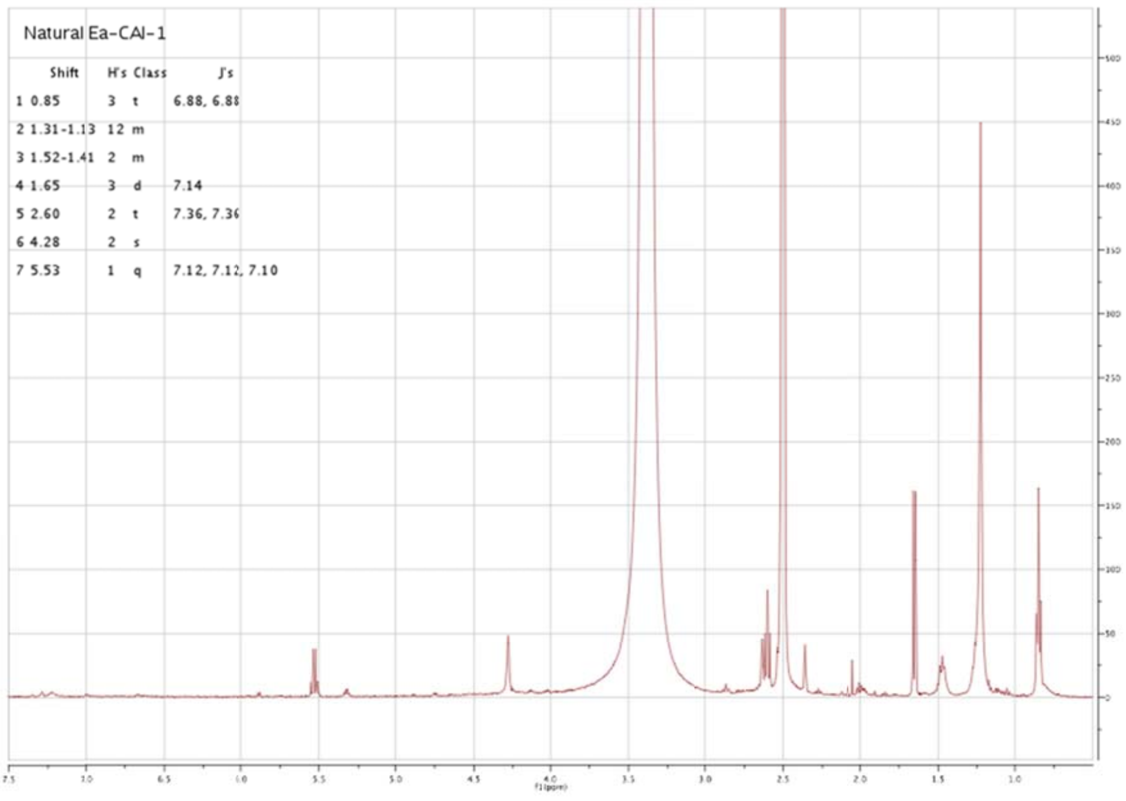
Natural Ea-CAI-1
Shift H's Class J's
1 0.85 3 t 6.88, 6.88
2 1.31-1.13 12 m
3 1.52-1.41 2 m
4 1.65 3 d 7.14
5 2.60 2 t 7.36, 7.36
6 4.28 2 s
7 5.53 1 q 7.12, 7.12, 7.10
f1 (ppm)

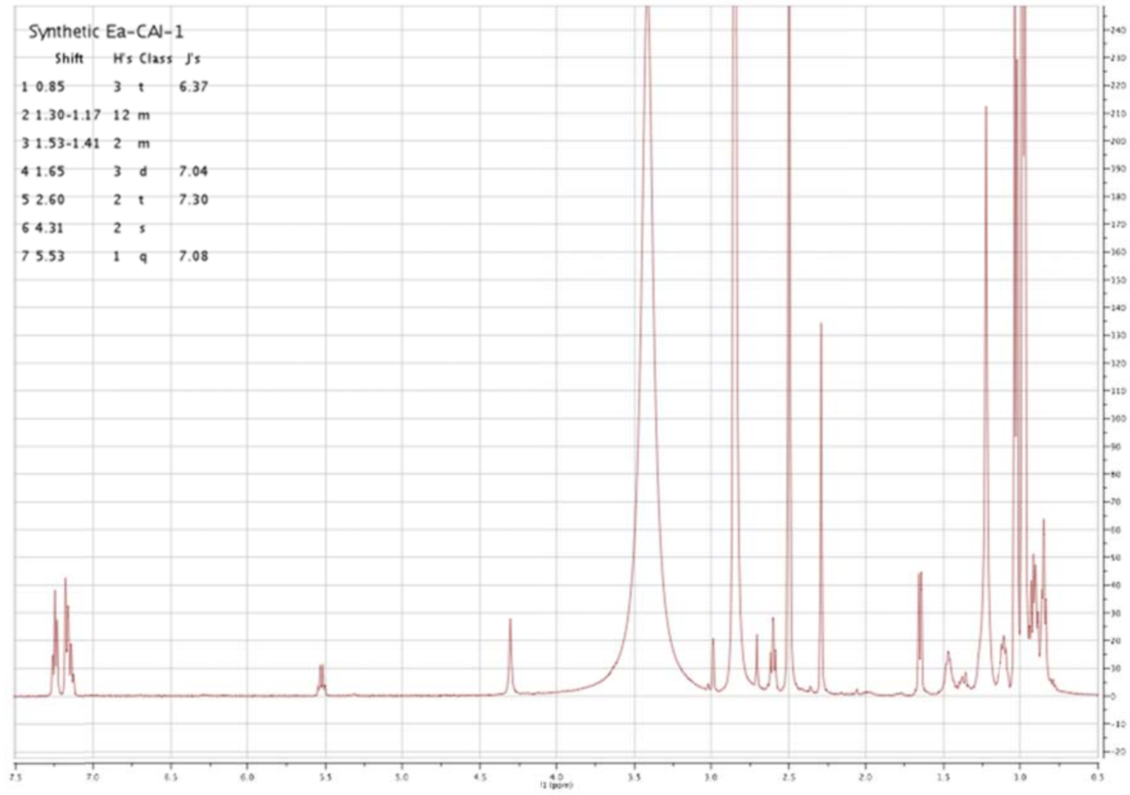
Synthetic Ea-CAI-1
Shift H's Class J's
1 0.85 3 t 6.37
2 1.30-1.17 12 m
3 1.53-1.41 2 m
4 1.65 3 d 7.04
5 2.60 2 t 7.30
6 4.31 2 s
7 5.53 1 q 7.08
f1 (ppm)

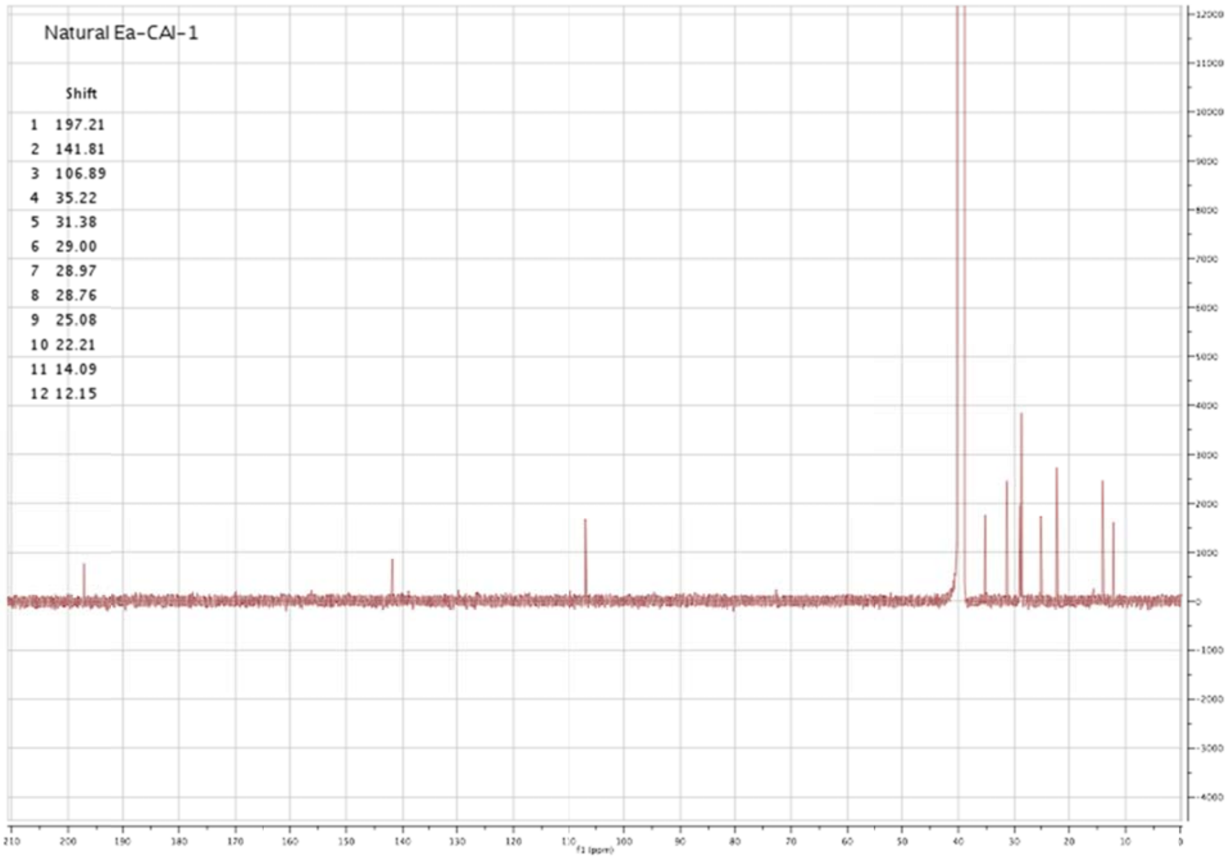
Natural Ea-CAI-1
Shift
1 197.21
2 141.81
3 106.89
4 35.22
5 31.38
6 29.00
7 28.97
8 28.76
9 25.08
10 22.21
11 14.09
12 12.15
f1 (ppm)

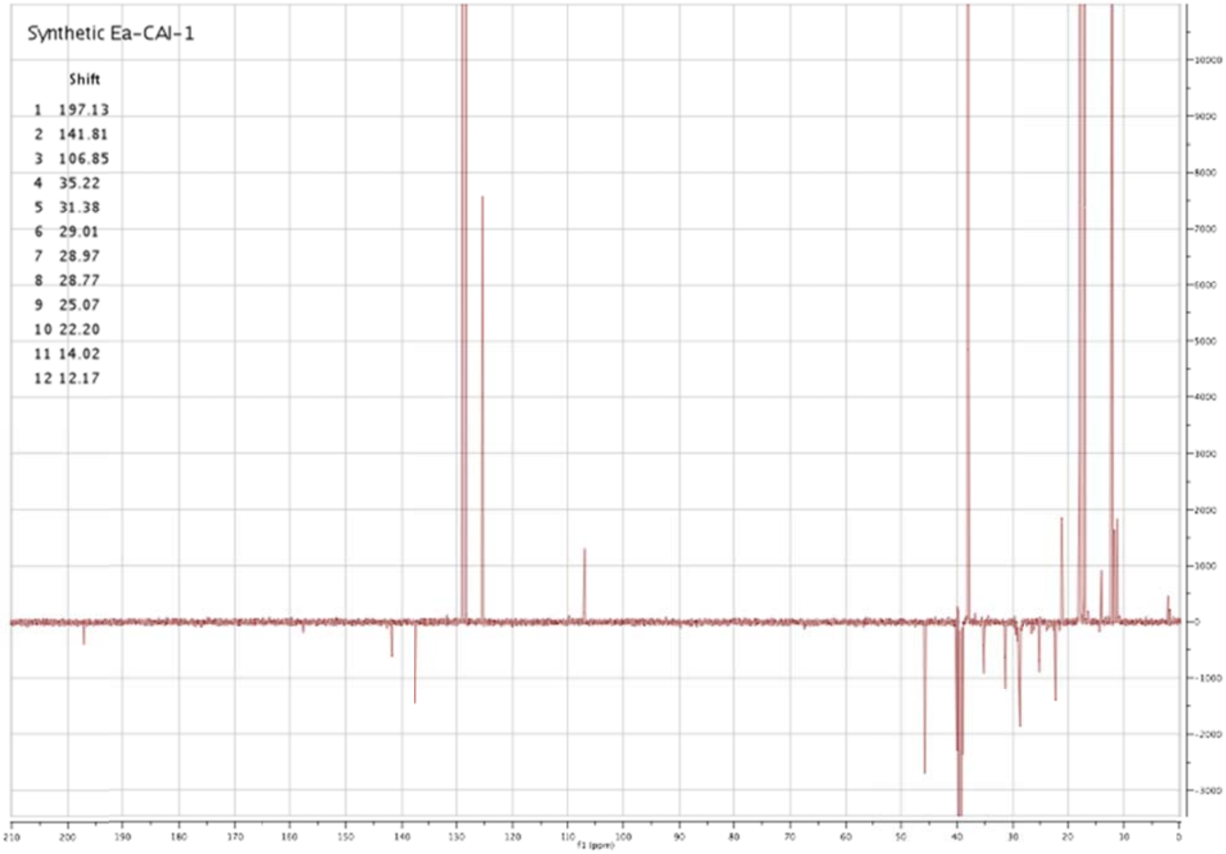
Synthetic Ea-CAI-1
Shift
1 197.13
2 141.81
3 106.85
4 35.22
5 31.38
6 29.01
7 28.97
8 28.77
9 25.07
10 22.20
11 14.02
12 12.17
f1 (ppm)

| **Synthetic Ea-CAI-1 $^{1}$H-NMR** | **Natural Ea-CAI-1 $^{1}$H-NMR** |
|---|---|
| **5.53 (q, *J* = 7.1 Hz, 1H)** | **5.53 (q, *J* = 7.1 Hz, 1H)** |
| **4.31 (s, 2H)** | **4.28 (s, 2H)** |
| **2.60 (t, *J* = 7.3 Hz, 2H)** | **2.60 (t, *J* = 7.4 Hz, 2H)** |
| **1.65 (d, *J* = 7.0 Hz, 3H)** | **1.65 (d, *J* = 7.1 Hz, 3H)** |
| **1.53-1.41 (m, 2H)** | **1.52-1.41 (m, 2H)** |
| **1.30-1.17 (m, 12H)** | **1.31-1.13 (m, 12H)** |
| **0.85 (t, *J* = 6.4 Hz, 3H)** | **0.85 (t, *J* = 6.9 Hz, 3H)** |

| **Synthetic Ea-CAI-1 $^{13}$C-NMR** | **Natural Ea-CAI-1 $^{13}$C-NMR** |
|---|---|
| **197.1** | **197.2** |
| **141.8** | **141.8** |
| **106.9** | **106.9** |
| **35.2** | **35.2** |
| **31.4** | **31.4** |
| **29.0** | **29.0** |
| **29.0** | **29.0** |
| **28.8** | **28.8** |
| **25.1** | **25.1** |
| **22.2** | **22.2** |
| **14.0** | **14.1** |
| **12.2** | **12.2** |

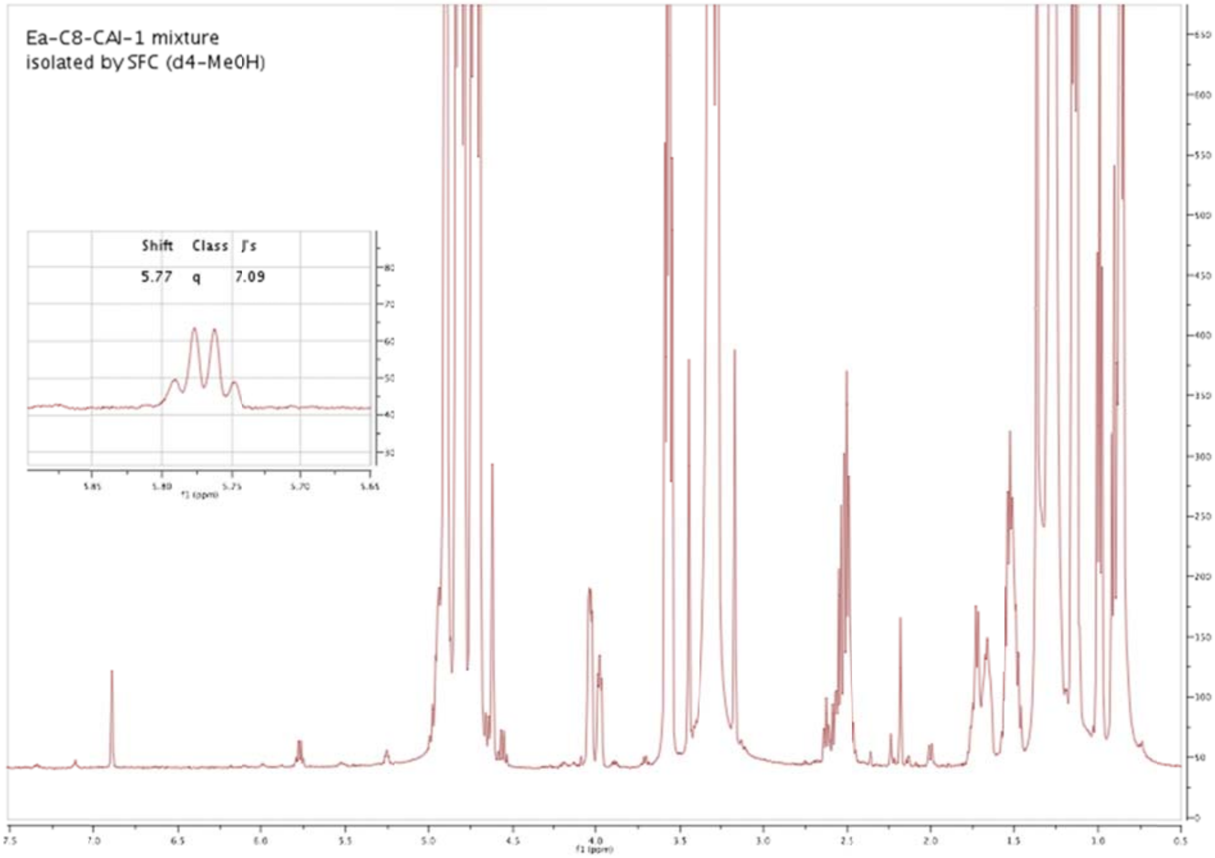
Ea-C8-CAI-1 mixture
isolated by SFC (d4-MeOH)
Shift Class J's
5.77 q 7.09
f1 (ppm)

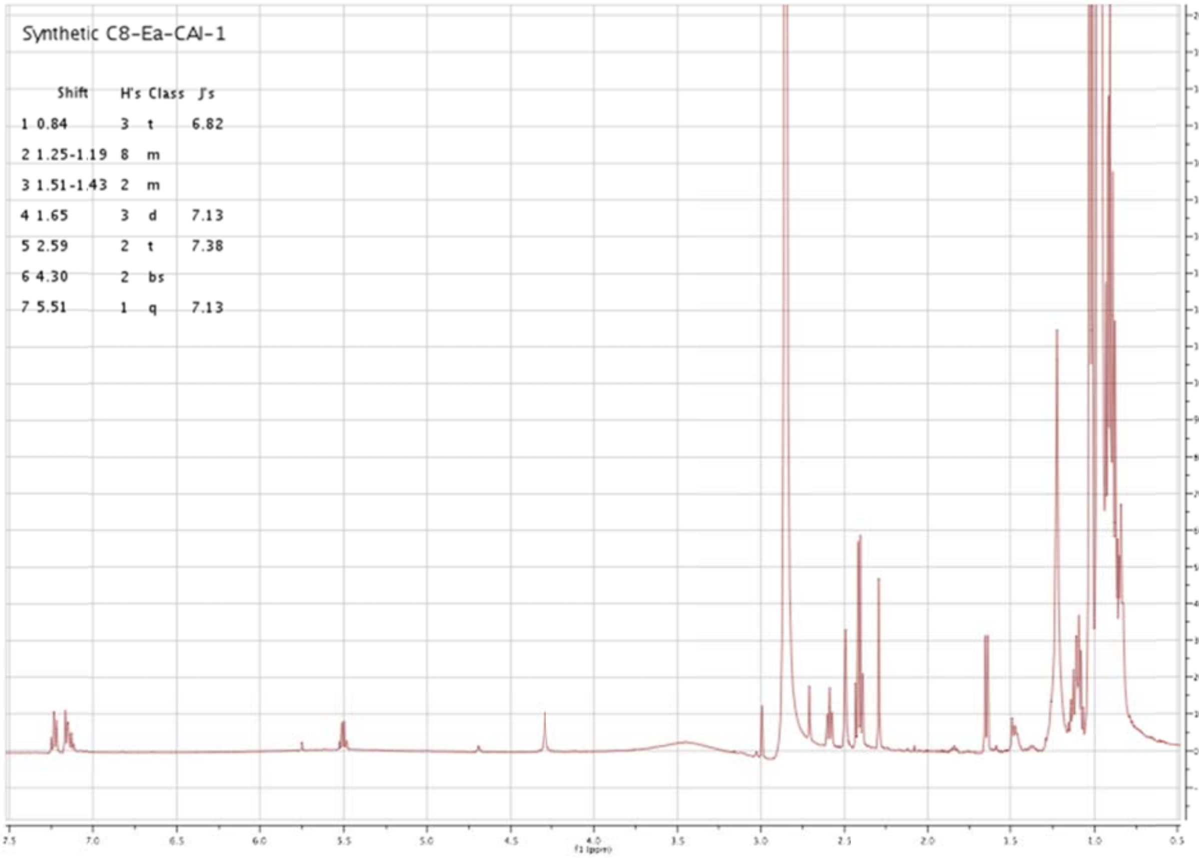
Synthetic C8-Ea-CAI-1
Shift H's Class J's
1 0.84 3 t 6.82
2 1.25-1.19 8 m
3 1.51-1.43 2 m
4 1.65 3 d 7.13
5 2.59 2 t 7.38
6 4.30 2 bs
7 5.51 1 q 7.13
f1 (ppm)

## References


Afzali-Ardakani, A., and Rapoport, H. (1980) L-Vinylglycine. *Journal of Organic Chemistry* 45: 4817.

Bassler, B.L., Greenberg, E.P., and Stevens, A.M. (1997) Cross-species induction of luminescence in the quorum-sensing bacterium Vibrio harveyi. *J Bacteriol* 179: 4043-4045.

Carrasco, M., Jones, R.J., Kamel, S., Rapoport, H., and Truong, T. (1992) N-(BENZYLOXYCARBONYL)-L-VINYLGLYCINE METHYL ESTER. *Organic Syntheses* 70: 29.

Friedman, A.M., Long, S.R., Brown, S.E., Buikema, W.J., and Ausubel, F.M. (1982) Construction of a broad host range cosmid cloning vector and its use in the genetic analysis of Rhizobium mutants. *Gene* 18: 289-296.

Henke, J.M., and Bassler, B.L. (2004) Three parallel quorum-sensing systems regulate gene expression in Vibrio harveyi. *J Bacteriol* 186: 6902-6914.

Higgins, D.A., Pomianek, M.E., Kraml, C.M., Taylor, R.K., Semmelhack, M.F., and Bassler, B.L. (2007) The major Vibrio cholerae autoinducer and its role in virulence factor production. *Nature* 450: 883-886.

Kelly, R.C., Bolitho, M.E., Higgins, D.A., Lu, W., Ng, W.L., Jeffrey, P.D., Rabinowitz, J.D., Semmelhack, M.F., Hughson, F.M., and Bassler, B.L. (2009) The Vibrio cholerae quorum-sensing autoinducer CAI-1: analysis of the biosynthetic enzyme CqsA. *Nat Chem Biol* 5: 891-895.

Miller, M.B., Skorupski, K., Lenz, D.H., Taylor, R.K., and Bassler, B.L. (2002) Parallel quorum sensing systems converge to regulate virulence in Vibrio cholerae. *Cell* 110: 303-314.

Ng, W.L., Wei, Y., Perez, L.J., Cong, J., Long, T., Koch, M., Semmelhack, M.F., Wingreen, N.S., and Bassler, B.L. (2010) Probing bacterial transmembrane histidine kinase receptor-ligand interactions with natural and synthetic molecules. *Proc Natl Acad Sci U S A* 107: 5575-5580.

Skorupski, K., and Taylor, R.K. (1996) Positive selection vectors for allelic exchange. *Gene* 169: 47-52.

Thelin, K.H., and Taylor, R.K. (1996) Toxin-coregulated pilus, but not mannose-sensitive hemagglutinin, is required for colonization by Vibrio cholerae O1 El Tor biotype and O139 strains. *Infect Immun* 64: 2853-2856.

Wei, Y., Perez, L.J., Ng, W.L., Semmelhack, M.F., and Bassler, B.L. (2010). *Submitted*.
